# Huntingtin CAG expansion impairs germ layer patterning in synthetic human 2D gastruloids through polarity defects

**DOI:** 10.1242/dev.199513

**Published:** 2021-10-05

**Authors:** Szilvia Galgoczi, Albert Ruzo, Christian Markopoulos, Anna Yoney, Tien Phan-Everson, Shu Li, Tomomi Haremaki, Jakob J. Metzger, Fred Etoc, Ali H. Brivanlou

**Affiliations:** 1 Laboratory of Stem Cell Biology and Molecular Embryology, The Rockefeller University, New York, NY 10065, USA; 2 Laboratory of condensed matter physics, The Rockefeller University, New York, NY 10065, USA

**Keywords:** Huntington's disease, Human embryonic stem cells, 2D gastruloids, TGFβ signaling, Epithelial polarity

## Abstract

Huntington's disease (HD) is a fatal neurodegenerative disorder caused by an expansion of the CAG repeats in the huntingtin gene (*HTT*). Although HD has been shown to have a developmental component, how early during human embryogenesis the HTT-CAG expansion can cause embryonic defects remains unknown. Here, we demonstrate a specific and highly reproducible CAG length-dependent phenotypic signature in a synthetic model for human gastrulation derived from human embryonic stem cells (hESCs). Specifically, we observed a reduction in the extension of the ectodermal compartment that is associated with enhanced activin signaling. Surprisingly, rather than a cell-autonomous effect, tracking the dynamics of TGFβ signaling demonstrated that HTT-CAG expansion perturbs the spatial restriction of activin response. This is due to defects in the apicobasal polarization in the context of the polarized epithelium of the 2D gastruloid, leading to ectopic subcellular localization of TGFβ receptors. This work refines the earliest developmental window for the prodromal phase of HD to the first 2 weeks of human development, as modeled by our 2D gastruloids.

## Abstract (precis)

**Summary:** 2D gastruloids of isogenic human embryonic stem cells modeling Huntington's Disease reveal that huntingtin CAG expansion perturbs the spatial restriction of the activin response in the context of the polarized epithelium.

## INTRODUCTION

Despite the fact that HD has been the first neurological disorder to be linked to a mutation in a single gene more than 25 years ago, neither the pathogenic mechanisms leading to neurodegeneration nor the normal functions of huntingtin (HTT) are well understood, and no therapy yet exists to treat or slow the progression of HD (Ross and Tabrizi, 2011). Although the *HTT* gene is ubiquitously expressed in all cells from the fertilized egg onwards, the expansion of CAG repeats predominantly causes degeneration of medium spiny neurons and layer V cortical neurons sometimes decades after birth. Contribution of animal models to understanding some aspects of HD neuropathology is hampered by the fact that they lack at least one human-specific HTT isoform, as well as human-specific attributes (Ruzo et al., 2015). Additionally, depending on the model system, tissue type or developmental stage, a large number of functions, ranging from vesicular transport (Elias et al., 2015; Gauthier et al., 2004), cell division (Elias et al., 2014; Ruzo et al., 2018), ciliogenesis (Haremaki et al., 2015), transcriptional regulation (Li et al., 2016) and autophagy (Martin et al., 2015) have been assigned to HTT. This pleiotropy of functions observed in different model systems, at different times and tissue types has made the understanding of molecular mechanisms in HD pathogenesis, and the distinction between cause and consequence very challenging.

Historically, CAG expansion has been considered to provide a gain of toxic function to HTT, mainly through aggregation of either the full-length or N-terminal fragments of mutant HTT (Ross and Tabrizi, 2011). However, a growing body of evidence is challenging this concept both *in vivo* and in hESC-based assays. Specific loss of HTT in subpallial lineages recapitulates HD pathology in mice (Mehler et al., 2019). Moreover, HTT^−/−^ phenocopies the results obtained from CAG-expanded lines in a self-organizing model of human neurula (Haremaki et al., 2019) and in hESC-derived neurons (Ruzo et al., 2018; Lopes et al., 2016). These data strongly suggest that, at least in this developmental context, CAG expansion is causing a loss of HTT function, rather than gain of toxic function.

Although the HD onset occurs relatively late in life, there has been a growing attention paid to the hypothesis of a developmental origin of HD, and the body of evidence that links early developmental defects with disease onset decades later in life is strengthening considerably. First, the most extreme mutations of *HTT* (longer than 54 CAG repeats) do lead to a juvenile form of HD that affects neurodevelopment. Second, although HD mutation carriers do not show any symptoms until the disease onset, there is evidence that individuals with HD present with specific changes decades before their diagnosis (Wiatr et al., 2018; Paulsen et al., 2010), including decreased ventricular volume. Third, developmental studies in stem cell-based systems, HD animal models as well as in HTT-CAG expanded human fetuses have clearly shown defects in cellular organization at early embryonic stages (Lo Sardo et al., 2012; Ruzo et al., 2018; Haremaki et al., 2019; Molero et al., 2009; Barnat et al., 2020). Finally, in mice, late disease phenotypes can be recapitulated if mutant HTT is present only soon after birth (Molero et al., 2016); conversely, there is evidence that early pharmacological treatment could be a promising therapeutic approach (Siebzehnrubl et al., 2018). Therefore, a crucial unanswered question remains in the HD field: when does the HD mutation produce its earliest effects during human embryogenesis?

In the human embryo, the earliest developmental transition occurring in the embryonic population (epiblast) is gastrulation, around day 14 post-fertilization: each cell differentiates towards one of the three germ layers and participates in a complex morphogenetic rearrangement leading to a three-dimensional layered organization (Arnold and Robertson, 2009). HTT is required for gastrulation, as *Htt*^−/−^ mice embryos show severe gastrulation defects and die at E7.5 (Woda et al., 2005; Zeitlin et al., 1995). The lack of studies reporting individuals with no HTT expression also points towards an early embryonic lethality in humans. In order to trace the developmental origin of HD, we took advantage of recent developments in synthetic embryology that allow the recapitulation of gastrulation *in vitro* from geometrically confined hESCs that self-organize into 2D gastruloids in response to BMP4. As this technology opens a unique door into the earliest events in human development, we have used our 2D gastruloid model to investigate whether mutant HTT would affect human gastrulation. Additionally, we use our collection of isogenic allelic series of hESCs engineered by CRISPR/Cas9 to incorporate different HTT repeat lengths that model the whole spectrum found in individuals with HD: the non-affected (20CAGs) and the adult-onset (43 and 48CAGs) patients up to the most extreme juvenile-onset patients (56 and 72CAGs) (Ruzo et al., 2018). The isogenicity of this allelic series, coupled to the highly quantitative model of human gastrulation, allows unprecedented sensitivity to identify mutant HTT-mediated defects at gastrulation stages. Comparative analysis of 2D gastruloids carrying different CAG lengths by precise analysis of patterning outcomes revealed a phenotypic signature for mutant HTT at gastrulation: a CAG-length dependent reduction in the ectodermal compartment, which is associated with an expansion in the endodermal population. This implies that mutant HTT can lead to embryonic defects as early as the second week of human development. Moreover, careful analysis allowed us to pinpoint the mechanism underlying the HD phenotype specifically to a polarity defect leading to alteration of the SMAD2 signaling pathway. This unveils the earliest deleterious effects of HD mutation, as detected in our models of the human gastrula.

## RESULTS

### CAG length-dependent HD phenotypic signature in human 2D gastruloids

**Fig. 1.**
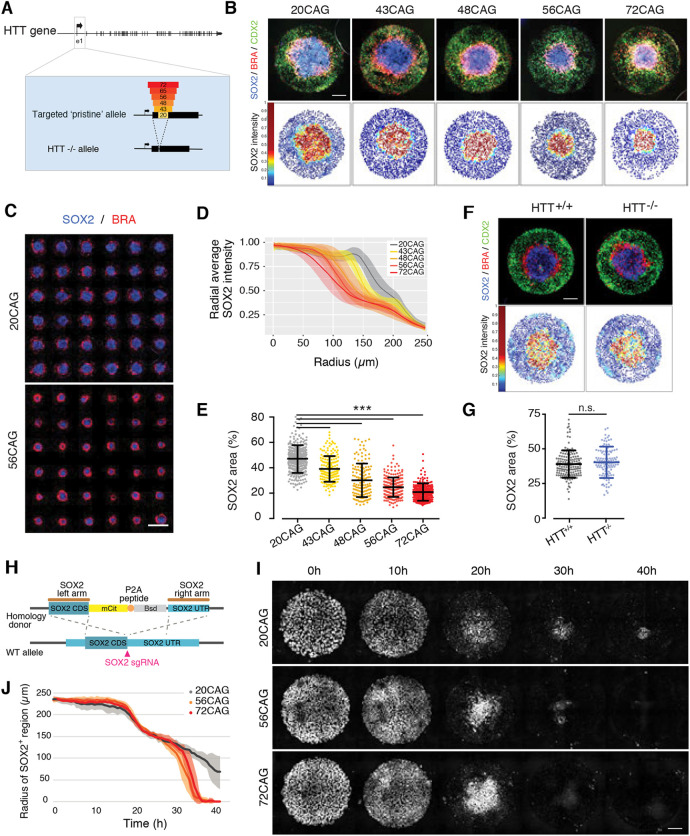
**CAG length-specific reduction of SOX2^+^ ectodermal lineage in 2D gastruloid differentiation.** (A) Generation of isogenic hESC collection using CRISPR/Cas9 genome engineering to model HD. The first exon of *HTT* was targeted and exchanged for increasing CAG lengths to create an allelic series or was deleted to generate *HTT*^−/−^. Three independent clones were isolated for each genotype. (B) Self-organization of CAG-expanded hESCs in geometrically confined micropatterns induced with BMP4 (50 ng/ml) in conditioned medium for 48 h. Concentric rings of ectodermal SOX2^+^ (blue), mesodermal BRA^+^ (red) and extra-embryonic tissue-like CDX2^+^ (green) domains probed by immunofluorescence (top). Dot plots (bottom) represent relative SOX2 intensity (red, high expression; blue, low expression) normalized to nuclear DAPI. Each dot corresponds to a single cell. (C) Family pictures of 20CAG control (top) and CAG-expanded 56CAG (bottom) 2D gastruloids. Micropatterns were induced with BMP4 and stained for SOX2 (blue) and BRA (red). (D) Analysis of mean SOX2 intensity across colonies with different CAG lengths using immunofluorescence data. Data are mean±s.d. of *n*>100 colonies for each genotype using *n*=2 or 3 clones. (E) Quantification of SOX2^+^ domain presented as area of total 2D gastruloid size (r=250 µm). Each dot corresponds to a single colony, plotted as mean±s.d. of *n*>100 colonies and *n*=2 or 3 clones/genotype. ***P<0.001, Kruskal–Wallis followed by Dunn's test. (F) BMP4-induced self-organization of *HTT*^−/−^ and *HTT*^+/+^ is visualized by immunostaining against SOX2^+^ (blue), BRA^+^ (red) and CDX2^+^ (green) domains (top). Spatial representation of SOX2 expression analyzed at single-cell resolution (bottom). (G) Analysis of SOX2^+^ area based on immunofluorescence data and plotted as a percentage of total 2D gastruloid size in *HTT*^+/+^ and *HTT*^−/−^. Data are mean±s.d. of *n*>100 colonies obtained using *n*=2 or 3 clones for each genotype. n.s. *P*>0.05, Mann–Whitney. (H) Strategy for generating mCitrine-SOX2 reporter lines in 20CAG, 56CAG and 72CAG genetic background using CRISPR/Cas9 technology. Coding region of SOX2 (SOX2 CDS) was labeled with mCitrine fluorescent tag (mCit) and separated from the blasticidin resistance gene (Bsd) by a P2A sequence. (I) BMP4-induced 2D gastruloid differentiation from time-lapse imaging series using mCitrine-SOX2 live reporter cell lines. Images shown before BMP4 addition (T=0 h) and every 10 h after stimulation in 20CAG, 56CAG and 72CAG. (J) Analysis of SOX2 radius as a function of time elapsed in control and HD 2D gastruloids (*n*=6). Images were acquired every 30 min. Individual colonies originate from a single micropatterned chip for each clone. Similar results were obtained from *n*=3 independent experiments. Scale bars: 100 µm.

We aimed to compare the differentiation pattern of HD isogenic lines (Fig. 1A) in our micropattern-based 2D gastruloid assay. Consistent with our earlier observations, after 48 h of BMP4 treatment, wild-type RUES2 colonies differentiated into 2D gastruloids containing spatially ordered specification domains with SOX2^+^ ectoderm, brachyury^+^ (BRA) mesoderm and CDX2^+^ extraembryonic tissue from center to edge (Figs 1B, S8). Under the same conditions, all HD isogenic hESC lines also induced the three embryonic germ layers, demonstrating that their differentiation potential was not affected. However, although the germ layers were induced similarly to the parental line, the radii associated with each germ layer ring were altered in the concentric circles (Fig. 1B). The analysis of several replicates per cell line revealed that the central ectodermal SOX2^+^ domain decreased in size proportionally to the increase in CAG length (Fig. 1C-E). Surprisingly, and in contrast to the CAG-expanded lines, HTT^−/−^ did not display a SOX2 reduction (Figs 1F,G, S8). To confirm the relationship between HTT-CAG expansion and reduced SOX2 area in an independent cell source, we used CAG-expanded induced pluripotent stem cells (iPSCs) generated from patient-derived fibroblasts. BMP4-induced 2D gastruloids of HD iPSCs displayed a reduction in their SOX2^+^ domain compared with the non-HD iPSC (Fig. S1C), similar to our hESC data. Furthermore, CRISPR/Cas9-correction of the HD mutation (Fig. S1A,B) rescued the SOX2 area to close to wild-type levels, demonstrating that the defect in ectodermal patterning is indeed caused by the CAG expansion (Fig. S1D-F). In order to track the dynamics of ectodermal fate acquisition, we generated SOX2 live-reporter lines using CRISPR/Cas9 gene-editing technology to fuse mCitrine to the C-terminus of SOX2 in 20CAG, 56CAG and 72CAG genetic background hESCs (Fig. 1H). As expected, the transcription factor SOX2 was homogeneously expressed in pluripotency, before BMP4 was added to the colonies (Fig. 1I). After stimulation with BMP4, the SOX2 signal remained high throughout the whole colony for 20 h, and then rapidly decreased at the colony edge (Fig. 1I,J). This is consistent with our previous demonstration that self-organization of 2D gastruloids follows a wave of differentiation that begins at the periphery and moves inwards (Warmflash et al., 2014; Etoc et al., 2016; Martyn et al., 2018). After 24 h, the SOX2 signal was solely localized at the colony center and gradually decreased in intensity. When comparing the parental 20CAG line with the CAG-expanded lines, no difference was observed during the first wave of differentiation. However, HD 2D gastruloids displayed a different dynamic of SOX2 expression: while the SOX2^+^ area of wild-type 2D gastruloids slowly retreated to the center of the colonies (Fig. 1I,J), HD 2D gastruloids displayed a much more abrupt reduction (Fig. 1J). This demonstrates that HTT mutation does not modify the early cell-intrinsic response to BMP4, but rather perturbs a process downstream of BMP4 signaling, which occurs 24 h post-stimulation.

### Mutant HTT enhances TGFβ signaling in 2D gastruloids at late differentiation times

**Fig. 2.**
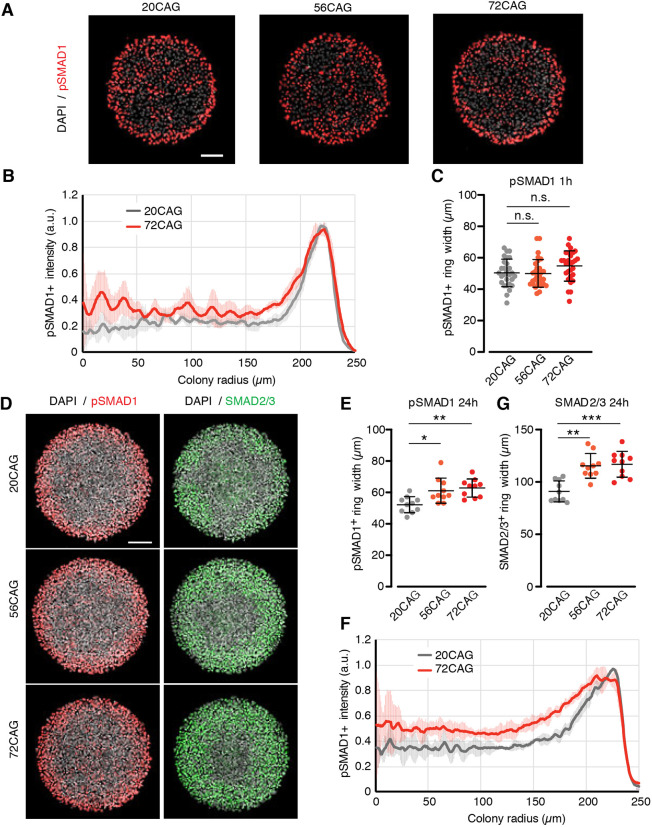
**CAG expansion leads to enhanced late pSMAD1 and SMAD2/3 response.** (A) Short-term BMP4 response (5 ng/ml) of 20CAG (left), 56CAG (middle) and 72CAG (right) in conditioned medium. Samples were fixed after 1 h of stimulation and immunostained for pSMAD1 (red); nuclei are visualized with DAPI (gray). (B) Mean radial pSMAD1 profile analyzed for the early response based on immunofluorescence data. Data are mean±s.d. of *n*>20 colonies/genotype. (C) Quantification of pSMAD1 activation from colony edge. The width of pSMAD1^+^ ring is determined based on radial intensity profile in early BMP4 signaling. Each data point corresponds to a single colony. Data are mean±s.d. of *n*>20 colonies/genotype. n.s. *P*>0.05, Kruskal–Wallis followed by Dunn's test. (D) Immunofluorescence data demonstrating activation of both TGFβ signaling branches after 24 h of BMP4 treatment (50 ng/ml) probed against the effectors pSMAD1 (red) and SMAD2/3 (green) in 20CAG (top), 56CAG (middle) and 72CAG (bottom). DNA is stained with DAPI (gray). (E) pSMAD1^+^ ring width in expanded CAG-length cell lines (56CAG and 72CAG) compared with 20CAG in late BMP4 signaling. Values are calculated from the radial intensity profile of individual colonies. Scatter plot of *n*=10 colonies/genotype. Data are mean±s.d. *P<0.05, **P<0.01, Kruskal–Wallis followed by Dunn's test. (F) Radial profiling of pSMAD1 intensity at 24 h. Data are mean±s.d. *n*>10 colonies/genotype. (G) 56CAG and 72CAG exhibit expanded nuclear SMAD2/3^+^ ring width compared with 20CAG. Width values of *n*=10 colonies/genotype. Data are mean±s.d. **P<0.01, ***P<0.001, Kruskal–Wallis followed by Dunn's test. Individual colonies originate from a single micropatterned chip for each clone. Similar results were obtained from *n*=3 independent experiments. Scale bars: 100 µm.

We have recently deciphered the molecular mechanism underlying the self-organization of germ layers in 2D gastruloids as the combination of two simple processes that are sufficient to quantitatively explain 2D gastruloid pattern formation: basolateral receptor re-localization and noggin (NOG) induction (Etoc et al., 2016). First, high cell density in the center of the colonies induces re-localization of TGFβ receptors to the basolateral side, thereby eliminating signal reception from the apically delivered ligand. Second, NOG, a secreted inhibitor, is induced with a concentration profile peaking at the center and lower at the edge. We therefore asked whether these two phenomena were affected by mutant HTT and could explain the CAG-dependent SOX2 domain shrinkage observed in HD 2D gastruloids. We compared the two longest repeat numbers available in our tool set, 56CAG and 72CAG, with a 20CAG control to capture the most severe effect of the mutation. In order to assess the efficiency of density-dependent receptor re-localization in the isogenic 2D gastruloids, we treated micropatterns with BMP4, and analyzed the distribution of phospho-SMAD1 across the colonies 1 h after ligand presentation. This early response is primarily mediated by the receptor localization component as it constitutes a too short timescale for diffusible inhibitors to be produced in high enough amounts (Etoc et al., 2016). As expected, cells at the periphery, exposing their receptors apically, were able to respond to BMP4. At this early time point, no differences were observed, the width of pSMAD1^+^ cells at the periphery were similar in wild-type and CAG-expanded colonies (Fig. 2A-C). This demonstrates that BMP4 signal reception is unaltered by the presence of mutant HTT.

We and others have shown that, during 2D gastruloid patterning, in a process closely mimicking the *in vivo* setting, BMP4 signaling induces WNT expression, which in turn activates the activin/NODAL pathway, a morphogen cascade that drives germ layer differentiation and organization (Martyn et al., 2018). We hypothesized that mutant HTT would affect this signaling crosstalk, resulting in patterning defects. As this signaling cascade needs time to build up (Warmflash et al., 2014), we turned our analysis to later time points of 2D gastruloid signaling and quantitatively assessed the status of BMP and activin/NODAL pathways by respectively staining for pSMAD1 and SMAD2/3 after 24 h of differentiation. As the pSMAD1 width is known to be density dependent, colonies with similar numbers of cells were analyzed (Fig. S2A). HD 2D gastruloids displayed a significant increase in the width of the pSMAD1^+^ ring at the edges, suggesting that mutant HTT is mediating an enhanced late BMP4 signaling (Fig. 2D-F). Moreover, HD 2D gastruloids displayed a wider SMAD2/3^+^ ring 24 h after differentiation than non-HD 2D gastruloids, suggestive of an increased signaling through the activin/NODAL pathway (Fig. 2G) at this time point. These results demonstrate that HTT-CAG expansion does not alter the initial BMP4 signal reception, but rather accelerate the differentiation, by enhancing both BMP and NODAL/activin signaling at later time points.

### Both CAG expansion and *HTT*^−/−^ enhance activin/NODAL signaling

**Fig. 3.**
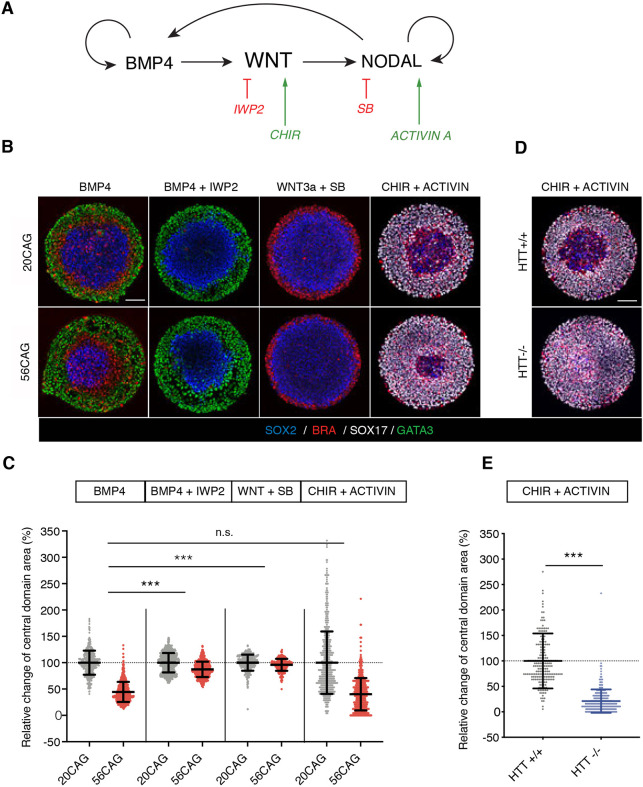
**Unraveling the effect of HTT-CAG expansion on the signaling events governing 2D gastruloid patterning.** (A) Schematic illustration of signaling hierarchy driving 2D gastruloid differentiation. BMP4 induces WNT, which in turn stimulates NODAL. Through a positive-feedback mechanism, BMP4 and NODAL also induce themselves. IWP2 is a pharmacological inhibitor of WNT secretion downstream of BMP4. CHIR 99021 is a chemical compound stimulating canonical WNT signaling cell-intrinsically. SB431542 selectively inhibits the activin/NODAL branch of TGFβ signaling. Activin acts through the same receptors and activates the same effectors as NODAL. (B) Dissection of 2D gastruloid signaling in 20CAG (top) and 56CAG (bottom). Three germ layers induced by BMP4 (50 ng/ml). IWP2 (2 µM) in combination with BMP4 (50 ng/ml) leads to abolished mesendoderm differentiation. In the absence of activin/NODAL signaling [blocked by SB431542 (10 µM)], WNT3a (100 ng/ml) induces mesoderm from the periphery. Activin (100 ng/ml) in combination with CHIR 99021 (6 µM) promotes endoderm differentiation in a radially symmetrical manner. Immunostaining was performed against SOX2 (blue), BRA (red), GATA3 (green) and SOX17 (gray) after 48 h of differentiation in conditioned medium. (C) The size of the central domain is determined as a relative change to the mean central domain area of 20CAG colonies as a percentage. Scatter plot represents normalized data points of *n*>250 colonies obtained from *n*=2 or 3 independent clones/genotype. Data are mean±s.d. in all respective conditions. Relative differences between the genotypes were statistically compared between each condition. n.s. *P*>0.05, ***P<0.001, ANOVA. (D) Fate acquisition in *HTT*^−/−^ compared with *HTT*^+/+^ in CHIR 99021 and activin-induced micropatterns probed by immunofluorescence against SOX2 (blue), BRA (red) and SOX17 (gray). (E) Quantification of the percentage change in central domain area relative to the mean central domain area of *HTT*^+/+^. Scatter plot represents normalized data points from *n*≥200 colonies/genotype. Data are mean±s.d. ***P<0.001, Mann–Whitney. Individual colonies were obtained from a single micropattern chip for each clone. Similar results were obtained from *n*=3 independent experiments in each condition. Scale bars: 100 µm.

In order to epistatically tie the patterning defect in CAG-expanded colonies to the signaling cascade underlying 2D gastruloid differentiation (Fig. 3A), independent nodes were systematically activated or inhibited. Patterning outcomes were evaluated by measuring the area of the central domain. In our standard 2D gastruloids, we observed a 55% reduction of the SOX2^+^ domain between 20CAG and 56CAG (Fig. 3B,C). Inhibition of WNT signaling by co-presentation of BMP4 with the small molecule inhibitor IWP2, led to 13% reduction of the SOX2^+^ center. This difference was further diminished by blocking activin/NODAL signaling using SB431542 downstream of BMP4 (Fig. S3D,E). Elimination of activin/NODAL downstream of WNT signaling by co-presentation of WNT3a and SB431542 showed 4% difference in SOX2^+^ area. Finally, cell-autonomous activation of the WNT pathway using CHIR 99021 coupled to SMAD2 activation via activin led to a dramatic 60% reduction in the central domain area (Fig. 3B,C, Fig. S3). Interestingly, the same co-treatment of CHIR 99021 and activin led to an even larger, 80%, reduction in the *HTT*^−/−^ (Fig. 3D,E). Overall, although an influence of WNT signaling cannot be completely excluded, this evidence demonstrates that HTT-CAG expansion has the strongest influence on the SMAD2 pathway not in the context of pluripotency maintenance, but during mesendodermal differentiation (Yoney et al., 2018). Moreover, loss of HTT further exacerbates this phenotypic signature, highlighting a previously unreported function of HTT as a necessary component of self-organization and specification of ectoderm in human 2D gastruloids, which is altered by the HTT-CAG expansion.

### CAG-expanded HTT selectively changes SMAD2 signaling dynamics without affecting transcriptional output

**Fig. 4.**
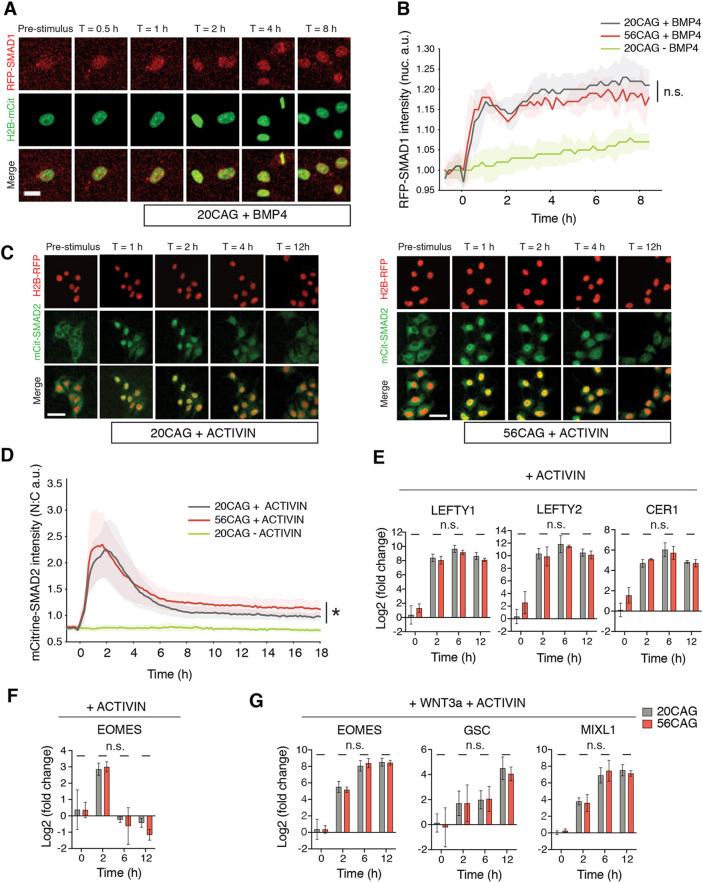
**HTT-CAG expansion selectively affects cell-autonomous SMAD2 signaling dynamics without altering transcriptional output.** (A) BMP4 (5 ng/ml) response of RFP-SMAD1 reporter single hESCs obtained from live imaging data in 20CAG pre and 0.5, 1, 2, 4 and 8 h post-ligand presentation in TeSR-E7 medium lacking TGFβ ligands. (B) Quantification of nuclear RFP-SMAD1 signal intensity in 20CAG, 56CAG and untreated 20CAG control single cells over time. Mean intensity values±s.d. are plotted for *n*>200 cells at each time-point in both genotypes. Cells were induced with BMP4 at T=0 h and imaged every 10 min. Similar results were obtained from *n*=2 independent experiments. n.s., *P*>0.05, unpaired *t*-test. (C) Response to activin (1 ng/ml) of 20CAG (left) and 56CAG (right) mCitrine-SMAD2 reporter single hESCs in defined TeSR-E7 medium. Snapshots from live imaging shown prior to and 1, 2, 4 and 12 h following stimulation. (D) Quantitative analysis of nuclear-to-cytoplasmic ratio (N:C) of mCitrine-SMAD2 intensities in single cells as a function of time. Data are mean signal intensities±s.d. of *n*>200 cells at each time-point are shown. Activin was added at T=0 h and images were acquired every 10 min in *n*=3 independent experiments. *P=0.0164, unpaired *t*-test. (E) Time-course RT-qPCR analysis of key pluripotency associated genes induced by SMAD2 upon activin (10 ng/ml) stimulation in both 20CAG (gray) and 56CAG (red) genotypes. (F) Expression of the SMAD2 target mesendoderm differentiation gene *EOMES* for 12 h following activin treatment. (G) SMAD2 target mesendoderm differentiation genes induced for over 12 h when stimulated with activin in combination with WNT3a (100 ng/ml). RT-qPCR analyses were performed in single cells using *n*=3 or 4 independent clones/genotype in defined TeSR-E7 medium and are plotted as mean±s.d. Data points obtained from *n*=4 technical replicates for each clone were normalized to internal GAPDH expression, then to the pre-stimulus (T=0 h) levels of each gene in 20CAG. n.s., *P*>0.05, unpaired *t*-test. Similar results were obtained from *n*=2 or 3 independent experiments in each condition. Scale bars: 25 µm in A; 50 µm in C. a.u. arbitrary units.

Having identified the activin/NODAL pathway as being responsible for the patterning defects observed in HD 2D gastruloids, we then turned towards investigating the molecular mechanism linking CAG-expanded HTT to enhanced SMAD2/3 signaling. The dynamics of TGFβ signaling in colonies can be divided into a cell-autonomous contribution, encompassing all intracellular events from receptor dimerization to downstream gene activation, and a non-cell autonomous contribution, involving the action of diffusible inhibitors and asymmetric ligand reception driven by cell polarization (Etoc et al., 2016). In order to distinguish at what level HTT-CAG expansion affects the SMAD pathway, we first tracked the dynamics of SMAD1 and SMAD2 nuclear translocation in single dissociated cells in response to ligand presentation. We knocked in a RFP-SMAD1 or a mCitrine-SMAD2 reporter into 20CAG control and 56CAG (Fig. S4A) using previously successful CRISPR/Cas9 strategy (Yoney et al., 2018). As shown in previous studies, when BMP4 was applied to single cell cultures of SMAD1 reporter lines, RFP-SMAD1 signal quickly accumulated in the nucleus in both genotypes and then remained stable (Fig. 4A; Yoney et al., 2018). In wild type and 56CAGs, measurement of nuclear RFP-SMAD1 translocation kinetics showed similar dynamics and intensity over time (Fig. 4B). We conclude that CAG expansion does not alter BMP4 signaling dynamics in single cells. Next, in order to evaluate the SMAD2/3 branch of TGFβ signaling, we compared the mCitrine-SMAD2 nuclear localization dynamics in single dissociated cells of 20CAG and 56CAG reporter cell lines after activin stimulation in defined serum-free culture conditions allowing for controlled TGFβ ligand levels. As reported previously and in contrast to the sustained RFP-SMAD1 response, mCitrine-SMAD2 displayed adaptive temporal dynamics in individual cells (Fig. 4C,D; Yoney et al., 2018). A pulse of nuclear localization was first observed during the first 6 h, followed by a stable accumulation to the nuclei with an elevated level compared with the pre-stimulus baseline (Yoney et al., 2018). The nuclear translocation of mCitrine-SMAD2 after stimulation with activin occurred at the same pace and same intensity in 20CAG and 56CAG hESCs during the first pulse of nuclear translocation. However, there was a small, but significant elevation in the long-term tail response (Fig. 4C,D). In order to probe whether this difference has a transcriptional consequence, we measured expression levels of SMAD2 target genes after activin presentation. We have previously shown that there are two types of activin-responsive genes: sustained pluripotency genes, such as *LEFTY1*, *LEFTY2* and *CER1*; and transient mesendodermal differentiation genes, such as *EOMES*. After activin treatment, no difference was detected in the induction levels of these genes in 20CAG and 56CAG backgrounds over time (Fig. 4E,F). Similarly, the same levels of expression of mesendodermal differentiation genes were measured when activin was presented with WNT3a (Fig. 4G). Therefore, HTT-CAG expansion affects cell-intrinsic SMAD2 signaling without affecting the transcriptional output, therefore the enhanced mesendodermal differentiation observed on the HTT-CAG expanded colonies cultured on micropatterns must arise downstream of cell-cell contacts.

### Both CAG expansion and *HTT*^−/−^ expand the spatial response to activin

**Fig. 5.**
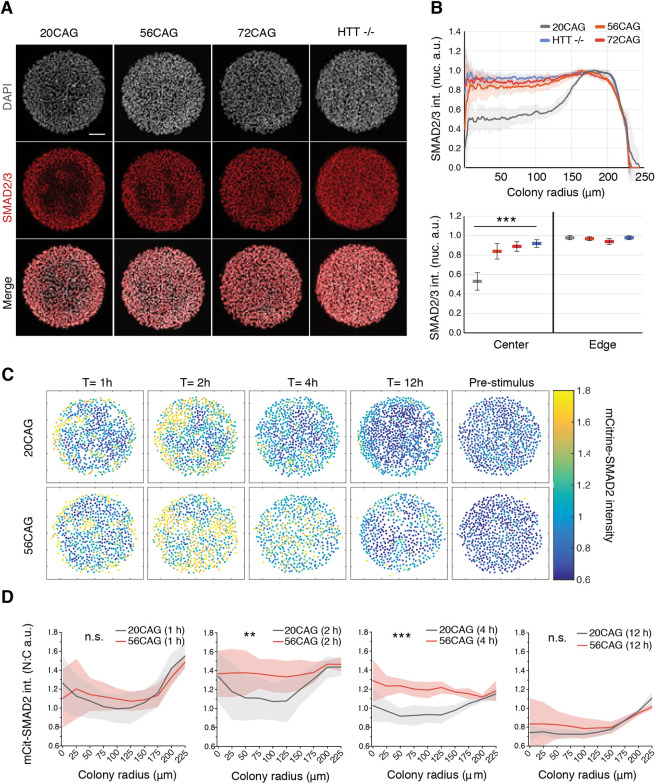
**HTT-CAG expansion and loss of HTT disrupt edge restriction in response to activin signaling.** (A) Early nuclear SMAD2/3 (red) accumulation probed by immunofluorescence with antibody recognizing total protein levels in response to activin (100 ng/ml) in conditioned medium. DAPI (gray) was used as a DNA stain. Samples were analyzed 1 h after induction. (B) Mean radial intensity profile of nuclear SMAD2/3 in 20CAG, 56CAG, 72CAG and *HTT*^−/−^. Error bars represent ±s.d. of *n*=15 colonies obtained from each genotype, all within a similar range of cell density (top). Mean nuclear SMAD2/3 intensity displayed in colony center versus colony edge in each genotype (bottom). ***P<0.001, Kruskal–Wallis followed by Dunn's test. (C) Temporal analysis of 20CAG and 56CAG mCitrine-SMAD2 reporter colonies before and 1, 2, 4 and 12 h after activin (10 ng/ml) treatment in defined TeSR-E7 medium. Cells were stimulated with activin at T=0 h. Dot plots represent mCitrine-SMAD2 intensity analyzed at a single-cell resolution. (D) Quantification of nuclear-to-cytoplasmic ratio (N:C) of mCitrine-SMAD2 intensities as a function of colony radius in 56CAG (red) compared with 20CAG (gray). Mean response±s.d. is calculated for *n*=6 (r=250 µm) colonies at 1, 2, 4 and 12 h after treatment with activin. n.s. *P*>0.05, **P<0.01, ***P<0.001, unpaired *t*-test. Individual colonies originate from a single micropatterned chip corresponding to each clone. Similar results were obtained from *n*=3 independent experiments. Scale bar: 100 µm. a.u. arbitrary units.

In order to identify the non-cell autonomous effect that is misregulated by both HTT-CAG expansion and *HTT*^−/−^, the dynamics of SMAD2 signaling in response to activin was explored at the colony level. We first quantified the early response of pluripotent colonies to activin, which is edge restricted due to basolateral localization of its receptors at the colony center (Etoc et al., 2016). Consistently, in 20CAG colonies, activin presentation led to the activation of a 80 µm ring of cells at the periphery (Fig. 5A,B). However, in 56CAG and 72CAG colonies, the SMAD2 signal was significantly extended towards the center. More dramatically, the *HTT*^−/−^ colonies completely failed to restrict the spatial response to activin, with the full colony responding homogeneously (Fig. 5A,B). In addition, and consistent with our hESC data, HD iPSCs failed to spatially restrict SMAD2/3 signaling in a CAG expansion-dependent manner (Fig. S5A,B). To elucidate the temporal dynamics of enhanced signal reception across genotypes, we evaluated mCitrine-SMAD2 nuclear translocation in colonies. 20CAG hESCs exhibited a pulse of mCitrine-SMAD2 translocation at the colony edge (Fig. 5C). In contrast, HTT-CAG expansion showed elevated nuclear mCitrine-SMAD2 localization in the center of colonies (Fig. 5C), as quantified in the radial profiles shown in Fig. 5D. Overall, these results suggest that wild-type HTT protein has an important function in maintaining a graded spatial response to activin, which is impaired by the HTT-CAG expansion and lost in *HTT*^−/−^.

### HTT-CAG expansion disrupts the polarized response to activin by affecting receptor relocalization

**Fig. 6.**
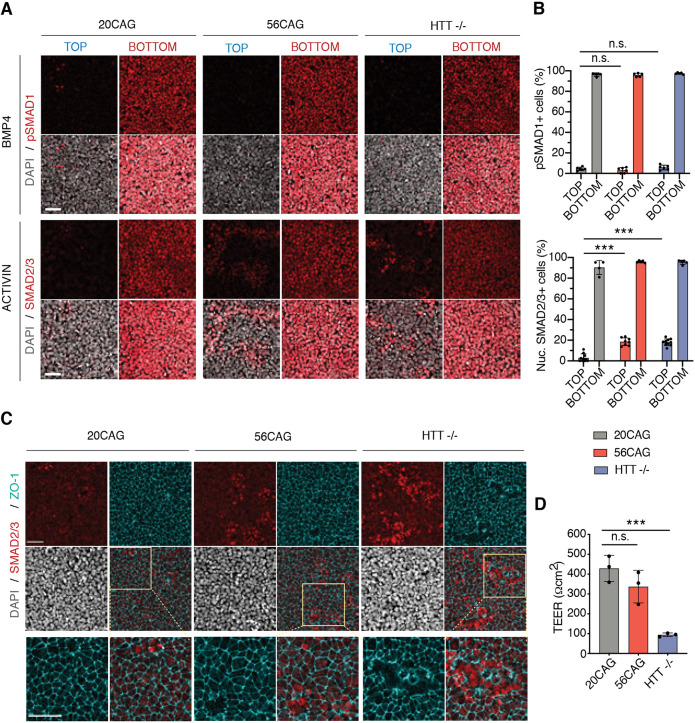
**HTT-CAG expansion disrupts the polarized activin response by affecting receptor relocalization.** (A) 20CAG, 56CAG and *HTT*^−/−^ hESCs grown on transwell inserts at high density are used to measure the apical versus basal BMP4 (10 ng/ml) response in conditioned medium. Immunofluorescence data for pSMAD1 (red) after ligand delivery to top and bottom compartments (top). Activin (10 ng/ml) induced nuclear SMAD2/3 (red) accumulation in 20CAG, 56CAG and *HTT*^−/−^ genotypes in defined TeSR-E6 medium probed by immunofluorescence (bottom). DNA visualized using DAPI (gray). Samples were analyzed 1 h after stimulation. (B) Percentage of pSMAD1^+^ cells (top) and nuclear SMAD2/3^+^ cells (bottom) across different HTT genotypes. Samples were analyzed at a single-cell resolution. The fractions of activated cells are presented as mean±s.d. of *n*≥5 images of apically and basally stimulated transwell filters. n.s. *P*>0.05, ***P<0.001, ANOVA. (C) Tight junction integrity was assessed in high-density hESC cultures on transwell filters by immunostaining against tight junction protein ZO-1 (cyan) at apical SMAD2/3 (red) activation sites in 20CAG, 56CAG and *HTT*^−/−^ followed by activin (10 ng/ml) stimulation in defined TeSR-E6 medium. Cell nuclei are visualized using DAPI (gray). Samples were analyzed 1 h after stimulation. (D) TEER was measured to determine epithelial integrity in 20CAG, 56CAG and *HTT*^−/−^. Data are mean±s.d. of *n*=3 independent measurements. n.s. *P*>0.05, ***P=0.001, ANOVA. Similar results were obtained from *n*=3 independent experiments. Scale bars: 50 µm.

**Fig. 7.**
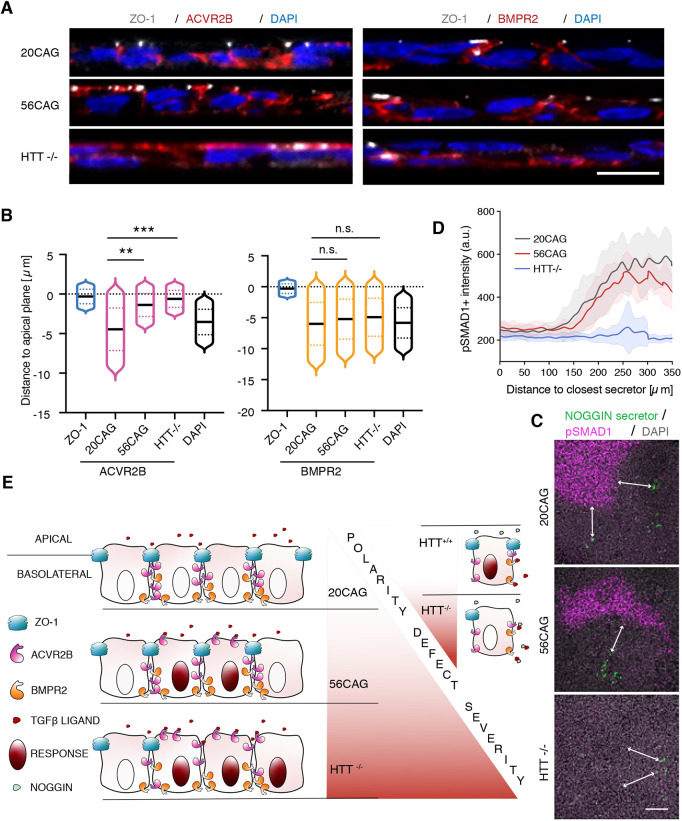
**Ectopic SMAD2/3 signaling is mediated by ACVR2B receptor mislocalization.** (A) Immunostaining of ZO-1 (gray) along with transiently expressed ACVR2B-HA (red, left) and BMPR2-HA (red, right) following doxycycline induction in 20CAG, 56CAG and *HTT*^−/−^. DNA is visualized using DAPI (blue). (B) Distance to apical plane defined by ZO-1 and measured for DAPI and ACVR2B (left) or BMPR2 (right) in 20CAG, 56CAG and *HTT*^−/−^. Histograms are represented as violin plots with each of their medians shown in black; dotted lines are 1st and 3rd quartiles. n.s. *P*>0.05, **P<0.01, ***P<0.001, Kruskal–Wallis followed by Dunn's test. (C) Immunostaining for pSMAD1 (magenta) and the H2B nuclear marker of doxycycline-induced NOG-expressing cells (green) after BMP4 treatment in 20CAG, 56CAG and *HTT*^−/−^. DAPI (gray) is used for DNA staining. Samples were fixed 16 h post-induction. Double-headed arrows indicate the distance between NOG-expressing and pSMAD1^+^ cells. (D) pSMAD1 intensity (a.u.) measured proximal to NOG-secreting cells in 20CAG, 56CAG and *HTT*^−/−^. Data are mean±s.d. for *n*≥15 NOG-secreting cells across *n*=8 images. (E) Schematic of the model by which CAG expansion disrupts polarity selective activin signaling and loss of HTT protein further compromises cellular polarity by impairing epithelial integrity in hESCs. Similar results were obtained from *n*=2 independent experiments. Scale bars: 10 µm in A; 100 µm in C. a.u.: arbitrary units.

We have shown previously that, in dense hESC epithelia, TGFβ receptors are localized to the basolateral side below the tight junctions insulating cells from apically delivered ligands (Etoc et al., 2016). Impaired spatial restriction to activin signaling observed in HTT-CAG expanded colonies suggests a failure of this mechanism. In order to functionally test this hypothesis, we used transwell filters to assess signaling response with either apical or basal TGFβ ligand presentation. As expected, pSMAD1 nuclear localization 1 h after BMP4 presentation demonstrated that wild-type hESCs respond to BMP4 only when applied to the basal compartment, and remain unresponsive when applied apically (Fig. 6A). Moreover, no difference in this polarized response to BMP4 was detected between 20CAG, 56CAG and *HTT*^−/−^ lines (Fig. 6B). Activin presentation to 20CAG line resulted in the same polarity-selective response as assessed by nuclear SMAD2/3 localization. However, 56CAG and *HTT*^−/−^ cells also responded to apically applied activin (Fig. 6A), with 20% of 56CAG and *HTT*^−/−^ cells showing ectopic nuclear SMAD2/3 localization. This ectopic response was always limited to a few cells dispersed across the filter in 20CAG (Fig. 6B, Fig. S6). This could come from a combination of two mechanisms: either the tight junction become leaky and let activin diffuse from the apical compartment to the basolateral side; or the activin receptors become mislocalized at the apical side. We therefore assessed tight junction integrity by examining ZO-1 integrity as well as the transepithelial electrical resistance (TEER) in our various genotypes. While 20CAG maintained an intact epithelium across the tissue, in the *HTT*^−/−^ line, ZO-1 expression was selectively absent in cells responding to stimulation with apically applied activin (Fig. 6C). Moreover, ectopic SMAD2/3 signaling observed in the 56CAG was not visibly accompanied by missing tight junction (Fig. 6C). Consistently, measuring TEER revealed that loss of HTT significantly impairs epithelial integrity and barrier function, but this is not the case in 56CAG (Fig. 6D). To elucidate how HTT-CAG expansion disrupts the polarized activin response, we used epitope tagged TGFβ type 2 receptors and visualized their localization in a doxycycline-inducible system (Etoc et al., 2016). As expected, 20CAG cells localized their activin-specific receptors to the basolateral surface. However, 56CAG and *HTT*^−/−^ left a significant fraction of the receptors exposed on the apical surface (Fig. 7A). Interestingly and despite the impaired tight junctions in the *HTT*^−/−^, BMPR2 localization was not affected in any of the genotypes (Fig. 7B). These results demonstrate that HTT is necessary for the maintenance of epithelial integrity. Moreover, both HTT-CAG expansion and loss of HTT selectively impair polarized activin signaling through inadequate and specific relocalization of the ACVR2B receptors to the basolateral surface.

### HTT-CAG expansion impairs polarity through loss of function

We demonstrated that loss of HTT elicits a range of polarity defects, including impaired epithelial barrier function and receptor mislocalization. This gives rise to ectopic activin signaling and leads to increased mesendodermal patterning. HTT-CAG expansion produces a similar, albeit less severe, phenotype than the *HTT*^−/−^ through a mechanism that is, at least in part, shared (Fig. 7E). Nonetheless, the intriguing question remains: if activin signaling is a key component of BMP4-induced patterning and the polarity of this pathway is severely affected by the loss of HTT, why are the consequences not apparent in the *HTT*^−/−^ 2D gastruloids? In addition to excluding the possibility of enhanced apoptosis (Fig. S7G,H), we wanted to test whether this was still the case under increased activin concentrations. We used BMP4 in combination with activin to induce 2D gastruloid differentiation and measured the size of the SOX2^+^ domain in *HTT*^−/−^. Interestingly, with elevated activin levels, the *HTT*^−/−^ was no longer able to maintain the unaffected 2D gastruloid patterning and produced a similar or even more severely reduced ectodermal domain to the HTT-CAG expanded cell lines (Fig. S7A-C). In contrast, blocked activin/NODAL signaling downstream of BMP4 led to an expansion of the SOX2^+^ domain in *HTT*^−/−^ compared with *HTT*^+/+^ (Fig. S7D-F). These results suggested that a compensatory mechanism specific to BMP4 induction must counterbalance the effect of endogenous activin/NODAL. Therefore, we used doxycycline-inducible expression to evaluate NOG reception in *HTT*^−/−^, with pSMAD1 serving as a proxy for signal attenuation (Phan-Everson et al., 2021). We found that, although the spatial range of NOG inhibition spanned over a 200 µm radius from the closest secretor cells in both 20CAG and 56CAG, this distance was markedly extended in *HTT*^−/−^ (Fig. 7C,D). Taken together, our results confirm that HTT-CAG expansion impairs germ layer patterning through polarity defects in activin signaling; this is consistent with a loss of function attributed to the HD mutation. Moreover, loss of HTT also affects the BMP branch of TGFβ signaling by enhancing signal inhibition.

## DISCUSSION

There is a growing body of literature about the prodromal symptoms of HD (Paulsen et al., 2014; Ciarochi et al., 2016; Harrington et al., 2015; Paulsen et al., 2008; Nopoulos et al., 2011) that affect the ventricular volume, among other features, of individuals carrying the mutation. At the most extreme end, studies demonstrating that embryogenesis is altered by the HD mutation are accumulating in stem cell-based assays (Haremaki et al., 2019; Ruzo et al., 2018; Lo Sardo et al., 2012; Conforti et al., 2018), as well as in animal models (Molero et al., 2016; Arteaga-Bracho et al., 2016; Siebzehnrubl et al., 2018). Moreover, in human embryos, HD mutation impacts development as early as gestational week 13 (Barnat et al., 2020). Here, using isogenic HD hESCs, we report that HTT-CAG expansion affects a model for human gastrulation. We have identified that germ layer specification defects mediated by HTT-CAG expansion are due to a positive modulation of SMAD2/3 signaling. In human 2D gastruloids, this led to enhanced mesendodermal differentiation and a smaller ectodermal domain, which ultimately gives rise to the central nervous system during development. Together with the reports of embryonic lethality of the *HTT*^−/−^ mice (Duyao et al., 1995; Zeitlin et al., 1995), our study provides, for the first time, evidence that HTT-CAG expansion could also affect gastrulation in humans. This constitutes the earliest developmental time point reported to be altered by the HD mutation. Interestingly, the severity of the phenotype directly correlates with the CAG lengths, similar to the onset of clinical symptoms and severity of disease progression.

It is important to highlight that our platform does not attempt to correctly recapitulate the symmetry breaking and body axis specification that occurs during gastrulation, rather it examines the signaling events controlling gastrulation in the context of an epithelium with conserved integrity in a highly reproducible and quantifiable manner. Therefore, an exploration of the impact of the HD mutation in recently developed 3D models of gastrulation would be complementary, as these systems soundly recapitulate body axis specification at the expense of epiblast epithelial integrity (Moris et al., 2020).

Our data unveil a possible mechanism underlying HTT-CAG expansion by pointing to a selective disruption in the polarity-dependent response to TGFβ ligands. Interestingly, our data draw a subtle picture for the disruption of cell polarity by the different genotypes: in the 56CAG background, whereas the response to BMP4 ligand was unaffected, activin signaling was significantly perturbed. Although homogeneous expression of the tight-junction protein ZO-1 on the apical side remained unchanged in the 56CAG background compared with the non-HD background, major patchy gaps of expression were detected in the *HTT*^−/−^ line. This could be a sign of a widespread and dynamic disruption of tight junction integrity, also characterized by the misplacement of other apical proteins such as Par, aPKC or others. In view of the importance of tight junction integrity in shaping the signaling gradients that pattern the mouse gastrula (Zhang et al., 2019), our study suggests a mechanistic explanation for the lethality observed in *HTT*^−/−^ mice and possibly in humans. As the 56CAG extended line phenocopies the *HTT*^−/−^ signature in ectopic activin response, this observation confirms that cellular polarization is compromised in the HTT-CAG expanded background, even though expression of ZO-1 remains intact. Although cell polarity has been extensively associated with HTT (Elias et al., 2015; Gauthier et al., 2004; Conforti et al., 2018), our study unveils the consequences of these defects at the molecular level, through selective impairments in TGFβ signal reception. Although we cannot fully exclude the possibility that signal propagation is also shaped by secreted inhibitors at later stages of these signaling events, the SMAD2/3 branch-specific effect could potentially be tied to differences in the targeting or localization of TGFβ, activin and BMP receptors. HTT plays an important role in endocytosis and its mutation affects endosomal recycling (McAdam et al., 2020; Moreira Sousa et al., 2013; Li et al., 2009). It has been shown that this can occur in a RAB11-dependent manner (Li et al., 2009), a mechanism that has been also implicated in the recycling of TGFβ receptors (Mitchell et al., 2004). Additionally, differences in the dynamics of TGFβ, activin and BMP receptor trafficking have been recently suggested (Miller et al., 2019). It is therefore possible that, in the context of polarized epithelium, HTT-CAG expansion selectively perturbs the sorting and recycling of TGFβ receptors specific to activin, but not to BMP. It would be interesting to evaluate whether these early changes elicited by the HTT-CAG expansion could contribute to premature or even trans-differentiation at later stages of development, downstream of gastruloid formation.

We and others have previously suggested that the HTT-CAG expansion leads to a loss of HTT function (Haremaki et al., 2019; Mehler et al., 2019; Ruzo et al., 2018). This is supported by a consistent set of observations that the HTT-CAG expanded lines phenocopy the *HTT*^−/−^ in a variety of assays. One seemingly exception to this rule is the lack of phenotype observed for the *HTT*^−/−^ in our BMP4-stimulated 2D gastruloid. This stands in striking contrast to the consistently strongest phenotypes observed in the *HTT*^−/−^ compared with expanded CAG backgrounds in the context of mesendodermal differentiation, SMAD2 activation and the polarity dependent defects in activin responses. Nonetheless, the activin concentration-dependence of this phenomena in *HTT*^−/−^ 2D gastruloids supports the notion of a compensatory mechanism that progressively fails under load. As a result, loss of HTT interferes with TGFβ signaling in two ways: it leads to enhanced activin reception and it also elevates BMP4 signal inhibition, thus canceling the SOX2 phenotypic signature in the corresponding 2D gastruloids. Both these mechanisms can be consequential to the widespread polarity defects displayed by the *HTT*^−/−^, thereby *HTT*^−/−^ might also disrupt the polarized targeting of NOG in addition to misplacing ZO-1 and its activin receptors. These results, contrary to the findings in the mouse, where loss of HTT impairs the specification and survival of mesendodermal progenitors, suggest a novel mechanism for *HTT*^−/−^ in the context of human gastrulation (Nguyen et al., 2013). Taken together, an overwhelming set of evidence points towards a graded loss of function of HTT that is dependent on CAG length and occurs during early development.

These results on the developmental component of HD, taken together with recent studies, suggests a revision of the binary view on HTT where the −/− genotype is embryonic lethal and the CAG expansion leads to neurodegeneration. These two observations, which are temporally segregated, can be tied together in an integrative model that includes neurodevelopment as a link. Currently, we do not know whether developmental defects cause clinical onset later in life, and recent studies have revealed normal motor and intellectual abilities of HD mutation carriers (Scahill et al., 2020). Moreover, development is a highly robust phenomenon, integrating complex compensatory mechanisms. Our study calls for more studies aimed at understanding how the embryo compensates for the defects mediated by HTT-CAG expansion. This may eventually open new clinical perspectives on preventive clinical interventions, aimed at fixing the underlying disease predispositions that are already in place before symptom onset, whether they involve the consequences of faulty development or homeostasis in the adult, or perhaps even sustain compensatory mechanisms that could delay clinical onset.

## MATERIALS AND METHODS

### Cell culture

hESCs and iPSCs were grown in HUESM conditioned with mouse embryonic fibroblasts and supplemented with 20 ng/ml bFGF (MEF-CM). Cells were maintained on tissue culture dishes coated with Geltrex (Thermo Fisher Scientific) and they were kept at 37°C with 5% CO_2_. Gentle Cell Dissociation Reagent and ReLeSR (Stemcell Technologies) were used for clump passaging. Cells were tested for *Mycoplasma* ssp. every 2 months.

### Generation of cell lines

The generation and validation of the human isogenic HD-RUES2 collection used in this study was previously reported by Ruzo et al. (2018). The reporter hESC lines were obtained using HDR-directed CRISPR/Cas9-mediated genome engineering. SMAD1 and SMAD2 were tagged with RFP and mCitrine on their N-termini, while the C-terminus of SOX2 was tagged with mCitrine. The fluorescent protein and the blasticidin or puromycin resistance cassette (BsdR or PuroR) were separated by a T2A self-cleaving peptide in the homology donors. CRISPR/Cas9 targeting was performed using the pX-335-based vector system (Cong et al., 2013) containing Cas9D10A nickase, including the chimeric guide RNA as previously published (Yoney et al., 2018; Martyn et al., 2018).

Human isogenic ESCs containing different lengths of CAG repeats in the *HTT* Exon1 locus (HD-RUES2; Ruzo et al., 2018) were nucleofected using the Cell Line Nucleofector II (Kit L from Lonza) by applying the B-016 program. Nucleofected cells were kept in MEF-CM supplemented with 10 µM ROCK inhibitor. Selection started 3-4 days later and was kept for 7 days in the case of blasticidin treatment and for 2 days in the case of puromycin treatment to select for targeted cells. Surviving cells were expanded and then passaged as single cells using Accutase (Stemcell Technologies) in MEF-CM supplemented with 10 µM ROCK inhibitor to grow clonal colonies. Colonies were expanded and genotyped using PCR amplification for the appropriate genomic region followed by Sanger sequencing. Validated targeted clones were subsequently transfected with ePiggyBac plasmids containing either H2B-mCitrine or H2B-mCherry cassettes to enable nuclear labeling for cell tracking (Lacoste et al., 2009). Individual clones were again isolated and controlled for normal karyotype (G-banding) and maintenance of pluripotency.

Cell lines expressing tagged TGFβ receptors and NOG under doxycycline control were generated as previously described (Etoc et al., 2016; Phan-Everson et al., 2021). HA tags were inserted on the C-terminal end of the BMPR2 and ACVR2B receptors, while NOG was tagged on the N-terminal end. ePiggyBac modified cell lines were generated using our standard nucleofection protocol and subsequently puromycin selected for 8-10 days. Doxycyclin was then applied to induce receptor expression 48 h or NOG expression 8 h prior to the experiment.

Patient-derived dermal fibroblasts were reprogrammed using Sendai viruses containing the Yamanaka factors according to the manufacturer's instructions (CytoTune-iPS 2.0 Sendai Reprogramming Kit, Thermo Fisher Scientific). iPSC colonies were identified by morphology and manually picked. Individual clones were expanded for 3 weeks and tested for normal karyotype (G-banding) and pluripotency maintenance. The iPSCs were then corrected for the HD mutation using CRISPR-Cas9 following the same methodology described by Ruzo et al. (2018). Individual clones were expanded and their genotypes were confirmed by PCR amplification of the appropriate genomic region followed by Sanger sequencing. Selected clones were further validated as reported above.

### Micropattern culture and 2D gastruloid differentiation

Prefabricated glass coverslips (Arena A, Cytoo) containing 500 µm disk patterns were coated with 10 µg/ml recombinant human laminin 521 (BioLamina) diluted in pre-warmed DPBS (Thermo Fisher Scientific) for 3 h at 37°C. Laminin was then serially washed with DPBS and coverslips were stored in a 35 mm tissue culture plastic submerged in the solution to prevent drying. hESCs were rinsed with DPBS-Mg/-Ca (Thermo Fisher Scientific) and dissociated to single cells with Accutase (Stemcell Technologies). 8×10^5^ cells were seeded on each coverslip in a defined volume of MEF-CM supplemented with 20 ng/ml bFGF (R&D Systems), 10 µM ROCK inhibitor (Y-27632, Abcam), 1× penicillin-streptomycin (Thermo Fisher Scientific) and 100 µg/ml Normocin (InvivoGen), and left unperturbed for 10 min to ensure homogenous distribution across the patterns. ROCK inhibitor was removed from the medium 3 h after seeding and cells were induced the following day with 50 ng/ml BMP4 (R&D Systems), 2 µM IWP2 (Stemgent), 10 µM SB431542 (Stemgent), 100 ng/ml WNT3a (R&D Systems), 6 µM CHIR99021 (EMD Millipore) and/or 100 ng/ml activin (R&D Systems). Fixed samples were analyzed by immunofluorescence 1, 24 or 48 h following induction.

### Transwell experiments

Transwell permeable polycarbonate membrane inserts (6.5 mm from Corning) were coated with 10 µg/ml recombinant human laminin 521 (BioLamina) diluted in DPBS (Thermo Fisher Scientific) for 3 h at 37°C and washed with DPBS twice. hESCs were passaged as single cells using Accutase (Stemcell Technologies) and seeded at 6×10^5^/cm^2^ or 3×10^5^/cm^2^ (corresponding to high or low cell density, respectively) on 24-well transwell plates in MEF-CM supplemented with 20 ng/ml bFGF (R&D Systems), 10 µM ROCK inhibitor (Y-27632, Abcam), 1× penicillin/streptomycin (Thermo Fisher Scientific) and 100 µg/ml Normocin (Invivogen). ROCK inhibitor was removed from the medium 3 h after seeding. Cells were stimulated with 10 ng/ml activin (R&D Systems) or BMP4 (R&D Systems) for 1 h the following day unless otherwise specified in the figure legends. To analyze SMAD2/3 response, cells were cultured in TeSR-E6 (Stemcell Technologies) lacking TGFβ ligand for 24 h prior to the experiment. After fixing, filters were removed from the transwell inserts, immunostained and mounted on a glass coverslip as described below. To assess transepithelial electrical resistance, cells were seeded at high density and resistance values were measured using a voltameter (EVOM2, World Precision Instruments) 48 h later. A laminin-coated well with no cells was used as a control. The average resistance of the control was subtracted from the measurement of each sample to calculate the tissue resistance per unit area.

### Live imaging

To follow RFP-SMAD1 and mCitrine-SMAD2 dynamics, hESCs or NPCs were seeded as single cells on optical plastic 35 mm dishes (ibidi) and cultured in appropriate growth medium supplemented with 10 µM ROCK inhibitor (Y-27632, Abcam) overnight. Before starting the experiment, cells were rinsed with DPBS (Thermo Fisher Scientific) to remove debris and switched to TesR-E7 imaging medium with added antibiotics (Yoney et al., 2018). Cells were kept in ROCK inhibitor throughout the experiment. The 2D gastruloid differentiation using mCitrine-SOX2 reporter lines was carried out in imaging quality MEF-CM supplemented with antibiotics. Live imaging was performed using spinning disk confocal microscope equipped with a 37°C incubation chamber supplied with 5% CO_2_, 445, 488 and 561 nm lasers and Hamamatsu 512×512 EMCCD camera (CellVoyager CV1000, Yokogawa). Images were acquired every 10 min for 24 h in the single cell experiments and for 50 h in the 2D gastruloid experiment using a 20×/0.75 NA objective lens in channels corresponding to RFP and mCitrine.

### Immunocytochemistry and imaging

Cells were fixed in 4% paraformaldehyde (Electron Microscopy Sciences) for 20 min, then washed three times with DPBS (Thermo Fisher Scientific) and permeabilized with 0.5% Triton X-100 (Sigma-Aldrich) in DPBS for 10 min. Samples were blocked with 3% normal donkey serum (Jackson ImmunoResearch) in PBST (0.1% Triton X-100 in DPBS) for 30 min at room temperature and incubated with primary antibodies (for antibody information and dilutions see Table S2, all of which were validated using negative controls and as stated on the manufacturer's website) diluted in blocking buffer overnight at 4°C, followed by three wash steps with PBST. Alexa Fluor 488-, 555- and 647-conjugated secondary antibodies (Invitrogen Molecular Probes, Thermo Fisher Scientific, 1:1000) were diluted in PBST along with DAPI nuclear counterstain (Thermo Fisher Scientific, 1:10,000) and samples were incubated for 1 h at room temperature. Two PBST washes and one DPBS wash were applied prior to mounting on a glass slide using ProLong Gold antifade reagent (Invitrogen Molecular Probes, Thermo Fisher Scientific). Tiled images of large areas were acquired with inverted wide-field epifluorescence microscope using 10×/0.45 NA objective lens (Zeiss Axio Observer Z1). Single *z*-stack confocal images were obtained with 20×/0.8 NA objective lens, using 405, 488, 561 and 633 nm laser lines, and a GaAsP detector (LSM 780, Zeiss).

### Image analysis

Quantification was carried out as described previously using custom software written in MATLAB (Etoc et al., 2016). In short, image tiles acquired from micropatterned culture experiments were stitched and background corrected. A foreground mask was created to detect colonies by thresholding the DAPI channel and calculating alpha shapes with respect to colony size. Each detected colony was extracted from the corrected and stitched image. The Ilastik classification tool was then used to segment individual cell nuclei. We filtered the DAPI image with parameters matching expected cell nucleus size to determine the center of each nucleus, which were used as seeds for subsequent watershed segmentation. A background mask was then applied to identify shapes of nuclei, and median nuclear intensities were extracted corresponding to each channel. To quantify SMADs or fate markers at a single-cell resolution in immunofluorescence samples, we classified the nuclear response as binary. A two-gaussian model was fitted on the distribution of normalized intensity values and Otsu thresholding was used to determine a cut-off between the two populations. Nuclei with intensity values above the threshold were considered positive. Based on the positional information of individual cells within the colonies, radial intensity profiles were created.

Experiments using the SMAD live reporters were analyzed similarly using the H2B image for nuclear segmentation as we described previously (Yoney et al., 2018). In the mCitrine-SOX2 live experiment, 6 colonies (500 µm in diameter; 683-785 cells/colony) were analyzed for each cell line. mCitrine-SOX2 nuclear signals were segmented on *z*-projected maximum intensity colonies using trainable weka segmentation. Radial intensity was calculated as mean pixel intensity as a function of distance from the colony center. We generated a graph showing the radial intensity every 30 min over the 50 h course of the experiment.

For the analysis of SOX2 area in immunostained 2D gastruloids, pixels were segmented into respective germ layer domains based on the joint histogram of intensity values. Otsu thresholding was used to minimize the weighted variance relative to the center of the three regions. Solid regions were delineated using alpha shapes and the areas of the domains were calculated.

Receptor localization was measured with respect to the apical surface determined by the ZO-1 stain (Etoc et al., 2016). Mean ZO-1 pixel intensity was calculated along the *z* axis for each *x*,*y* coordinate to interpolate a continuous apical surface. Then mean pixel intensities along the *z* axis were measured for each *x*,*y* position in all subsequent channels and plotted with respect to the apical surface.

### RT-qPCR

10^4^ cells/cm^2^ were seeded as single cells in defined TeSR-E7 medium (Stemcell Technologies) lacking any TGFβ ligands and supplemented with 10 µm ROCK inhibitor (Y-27632, Abcam). Cells were stimulated the following day with either activin (10 ng/ml) alone or in combination with WNT3a (100 ng/ml) and collected using Trizol (Life Technologies) 0 (pre-stimulus), 2, 6 and 12 h later. Total RNA was isolated from each sample using RNeasy Mini Kit (QIAGEN) and cDNA was synthesized using Transcriptor First Strand cDNA Synthesis Kit (Roche). qPCR was performed in SYBR Green I Master Mix with sequence specific primers for genes of interest (Table S1) in four technical replicates at 55°C annealing temperature for 45 cycles using Lightcycler 480 instrument. GAPDH was used for internal normalization.

### Study design and statistical analysis

All data presented in this study were obtained from at least two independent experiments. Micropatterns and qRT-PCR experiments were carried out using multiple biological replicates. Key phenotypes found in this study were replicated and confirmed in an independent cell source. Sample size was determined based on previous experiences. In micropatterned cell culture, merged colonies or colonies that could not be well isolated were excluded from the analysis as established in previous studies. No particular randomization method was used and no blinding was performed in this work. Statistically significant differences between two conditions were determined using an unpaired two-tailed *t*-test or a two-sided Mann–Whitney test. Multivariate comparisons were performed using Kruskal–Wallis followed by Dunn's test; alternatively, Dunnett's (one-way ANOVA) or Tukey's method (two-way ANOVA) was used. Statistical analyses were performed in Prism 8 (GraphPad). Results were not significant (n.s.) or *P<0.5, **P<0.01 and ***P<0.001, unless otherwise specified.

### Data and code availability

The datasets generated and analyzed in this study are available upon reasonable request from the corresponding authors. The image analysis software used in this study are available upon request from the corresponding authors.

### Ethics statement

All hESC experiments were performed using genetically engineered clones derived from the RUES2 (NIH #0013) parental cell line, which was created in our lab and is listed in the NIH Human Embryonic Stem Cell Registry. Human iPSCs were generated from patient-derived fibroblasts. The cell lines used in this study were derived and genetically manipulated under the approval from the Tri-Institutional Stem Cell Initiative Embryonic Stem Cell Research Oversight (Tri-SCI ESCRO) Committee, an independent committee charged with oversight of research with human pluripotent stem cells and embryos to ensure conformance with University policies and guidelines from the US National Academy of Sciences (NAS) and International Society for Stem Cell Research (ISSCR).

### Cell lines and clones

RUES2-HD allelic series used in this study was characterized and published previously (Ruzo et al., 2018). Additional control clones 20CAG-4 and 20CAG-5 were derived subsequently and used in Fig. 3B and Fig. 4E-G following the same validation process as described for the RUES2-HD allelic series. RFP-SMAD1 and mCitrine-SMAD2 live reporters used in Fig. 4A-D and Fig. 5C,D were generated from 20CAG-2, 56CAG-3 clones. mCitrine-SOX2 live reporters used in Fig. 1H-J were derived from 20CAG-1, 56CAG-3 and 72CAG-1 clones. HD-1 and HD-2 HTT-CAG expanded iPSC clones were corrected for the mutation and named HD-C1 and HD-C2. Unaffected fibroblasts were reprogrammed to Non-HD iPSCs.

## Supplementary Material

Supplementary information

Reviewer comments
