# Supplementary material for: Huntingtin CAG expansion impairs germ layer patterning in synthetic human 2D gastruloids through polarity defects
Source: Development. 2021 Oct 5;148(19):dev199513. doi: 10.1242/dev.199513 (PMC8513611; doi:10.1242/dev.199513)
Supplement: Supplementary information [file develop-148-199513-s1.pdf]

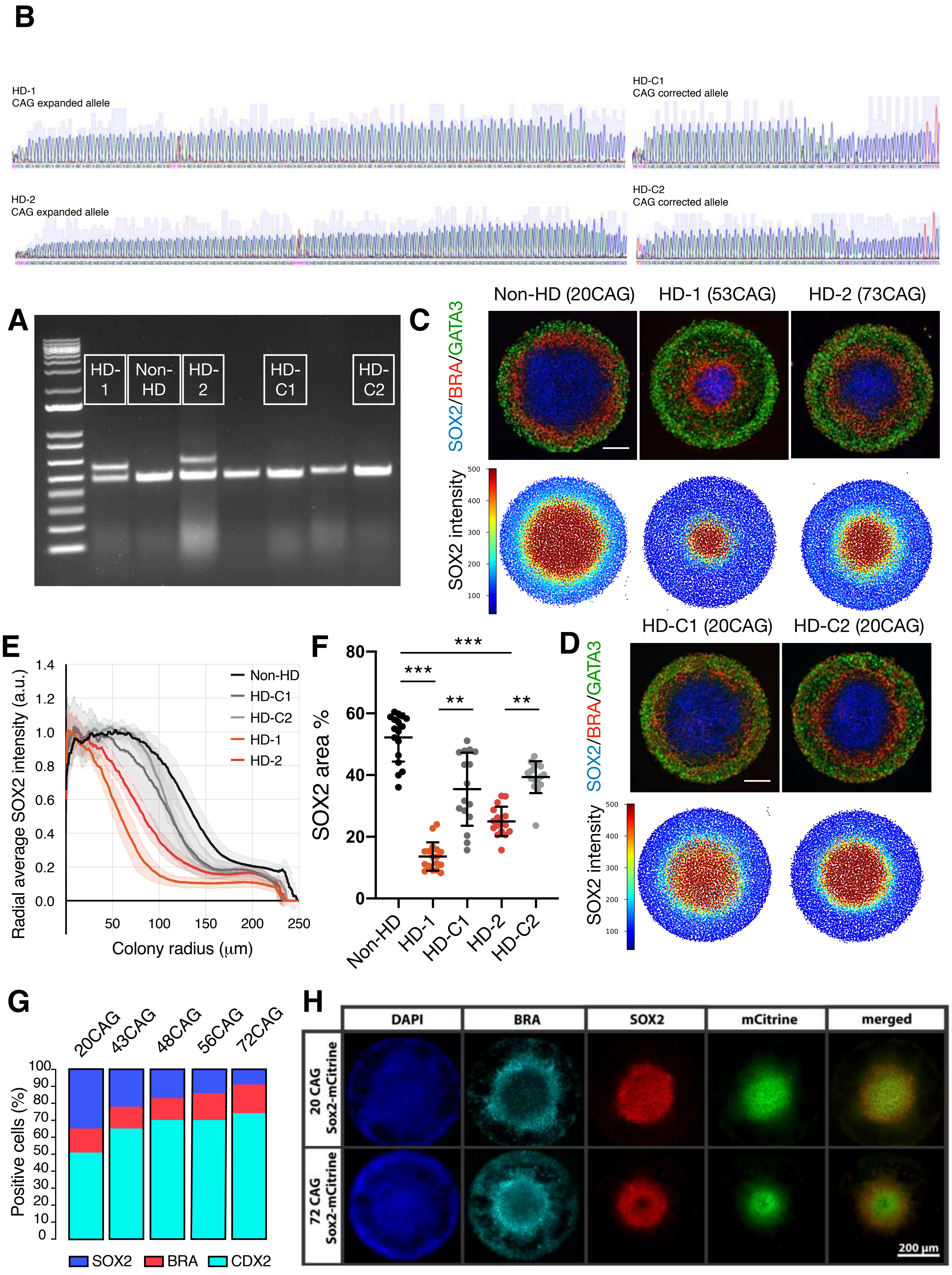

Development • Supplementary information

Fig. S1.

Characterization of mCitrine-SOX2 live reporter and HD iPSCs in 2D gastruloid differentiation.

A. PCR gel of Non-HD and HD iPSCs with their CRISPR-corrected counterparts.

B. Sanger sequencing of the CAG expanded (left) and CAG corrected (right) alleles of HD iPSCs.

C. BMP4-induced (50 ng/ml) 2D gastruloids of Non-HD (left) and HD (middle and right) iPSCs immunoprobed by SOX2 (blue), BRA (red) and GATA3 (green) after 48 hours of differentiation in conditioned medium (top). Relative SOX2 intensity (red=high, blue=low expression) in composite dot plots of n>15 colonies for each genotype (bottom panel). Each dot represents a single cell.

D. BMP4-induced (50 ng/ml) 2D gastruloids of CRISPR-corrected HD iPSCs. Immunostaining shown for SOX2 (blue), BRA (red) and GATA3 (green) in top panel. Relative SOX2 intensity is shown in composite dot plots of n>15 colonies for each cell line in bottom panel as described previously.

E. Radial mean SOX2 intensity of Non-HD and HD iPSCs with CRISPR-corrected counterparts based on immunofluorescence data. S.d. is calculated from n>15 colonies for each clone.

F. SOX2 area of each 2D gastruloid represented as percentage of total 2D gastruloid size (r=250 µm). Each dot corresponds to a single colony for each of the clones. ***p<0.001, **p<0.01, Kruskal-Wallis followed by Dunn.

G. Percentage of positive cells in each of the germ layer domains in HD-hESC colonies corresponding to Figure 1B.

H. BMP4-induced (50 ng/ml) 2D gastruloid of mCitrine-SOX2 cell line. Sample was fixed and stained after 48 hours of differentiation. DAPI (blue), BRA (cyan), SOX2 (red). mCitrine-SOX2 live reporter is shown in green.

Scale bars: 100 µm (C, D), 200 µm (G).

a.u. arbitrary units

Development • Supplementary information

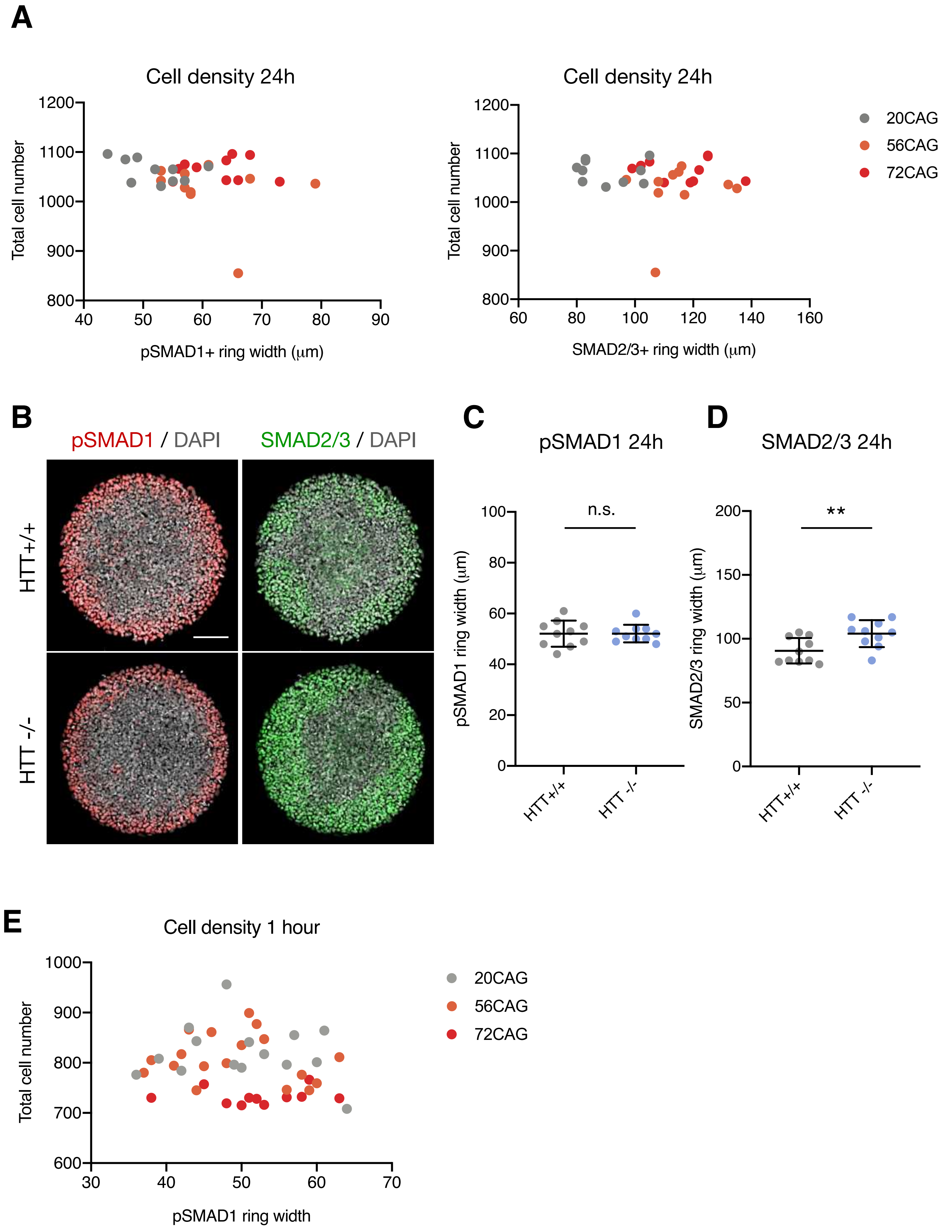

Development • Supplementary information

Fig. S2.

The effect of cell density and loss of HTT on TGFβ signaling in BMP4-induced 2D gastruloids.

- **A.** pSMAD1+ (left) and SMAD2/3+ (right) ring width values 24 hours post BMP4 induction plotted in the function of cell density for 20CAG, 56CAG and 72CAG colonies. Total cell numbers were calculated for each of the colonies corresponding to the dots (n=10).
- **B.** Immunofluorescence data of $HTT^{+/+}$ and $HTT^{-/-}$ displaying pSMAD1 (red) and SMAD2/3 (green) as a composite image with DAPI (gray) 24 hours following BMP4 induction.
- **C.** pSMAD1+ ring width determined based on mean radial intensity of immunofluorescence data presented in panel B. Each data point represents a single colony with mean ±s.d. of n=10 colonies. n.s. $p>0.05$, Mann-Whitney.
- **D.** SMAD2/3+ ring width calculated from mean radial intensity of immunofluorescence data presented in panel B. Each data point represents a single colony with mean ±s.d. of n=10 colonies. ** $p<0.01$, Mann-Whitney.
- **E.** pSMAD1+ ring width in the function of cell density at 1 hour following BMP4 stimulation in 20CAG, 56CAG and 72CAG. Total cell numbers were calculated for each of the colonies that correspond to each of the dots (n>10).

  Scale bar: 100 µm.

Development • Supplementary information

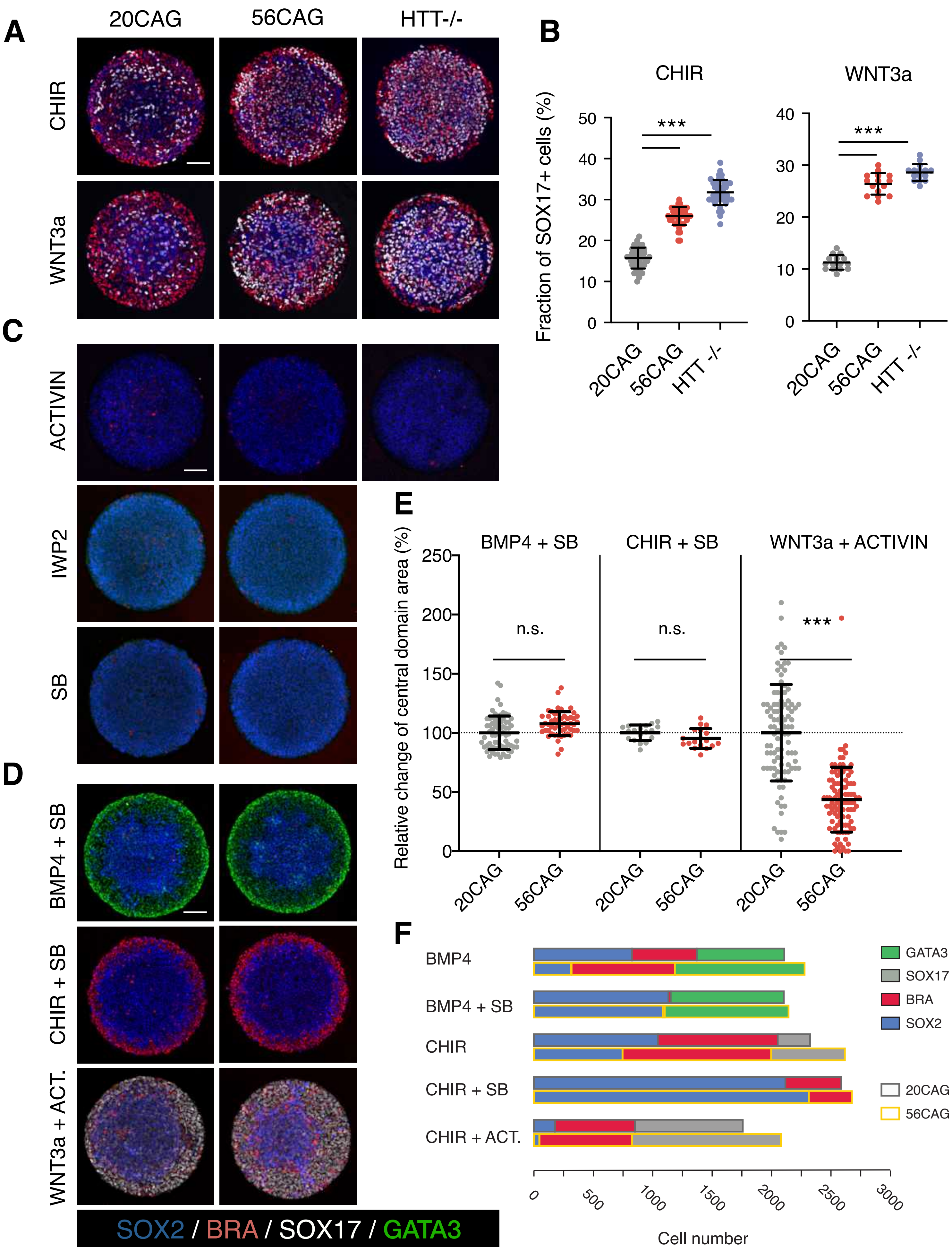
A
20CAG
56CAG
HTT-/-
CHIR
WNT3a
B
CHIR
WNT3a
Fraction of SOX17+ cells (%)
***
***
20CAG
56CAG
HTT -/-
C
ACTIVIN
IWP2
SB
D
BMP4 + SB
CHIR + SB
WNT3a + ACT.
SOX2 / BRA / SOX17 / GATA3
E
BMP4 + SB
CHIR + SB
WNT3a + ACTIVIN
Relative change of central domain area (%)
n.s.
n.s.
***
F
BMP4
BMP4 + SB
CHIR
CHIR + SB
CHIR + ACT.
GATA3
SOX17
BRA
SOX2
20CAG
56CAG
Cell number

Fig. S3.

The effect of activators and inhibitors of early embryonic signaling on micropatterned colonies.

- **A.** Comparison of 20CAG, 56CAG and $HTT^{-/-}$ after 48 hours of differentiation in conditioned medium using CHIR 99021 (6 μM) or WNT3a (100 ng/ml). Samples were fixed and immunostained against SOX2 (blue), BRA (red), SOX17 (gray).
- **B.** Endodermal differentiation assessed by the fraction of SOX17+ cells in each genotype downstream of WNT signaling induced by CHIR 99021 or WNT3a. Each dot represents a single colony, mean ±s.d. of n>15 colonies. ***p<0.001, Kruskal-Wallis followed by Dunn.
- **C.** Colonies homogeneously express SOX2 (blue) with a few BRA+ (red) cells interspersed following ACTIVIN (100 ng/ml), SB431542 (10 μM) or IWP2 (2 μM) induction in the genotypes tested.
- **D.** SB431542 (10 μM) with BMP4 (50 ng/ml) leads to abolished mesendodermal differentiation, while it hinders endodermal differentiation in combination with CHIR 99021 (6 μM). WNT3a (100 ng/ml) in combination with ACTIVIN (100 ng/ml) induces radially symmetrical self-organization in both genotypes from colony edge to center in 20CAG and 56CAG. Immunostaining was performed against SOX2 (blue), BRA (red), SOX17 (gray) and GATA3 (green).
- **E.** Relative change in central domain area compared to the mean area of 20CAG central domain in each of the conditions. Each dot represents a single colony in the scatter plots with mean ±s.d. of n≥20 colonies. n.s. p>0.05, ***p<0.001, Kruskal-Wallis followed by Dunn.
- **F.** Comparison of cell numbers corresponding to each of the germ layer domains of representative colonies under various conditions in 20CAG and 56CAG.

  Scale bars: 100 μm.

Development • Supplementary information

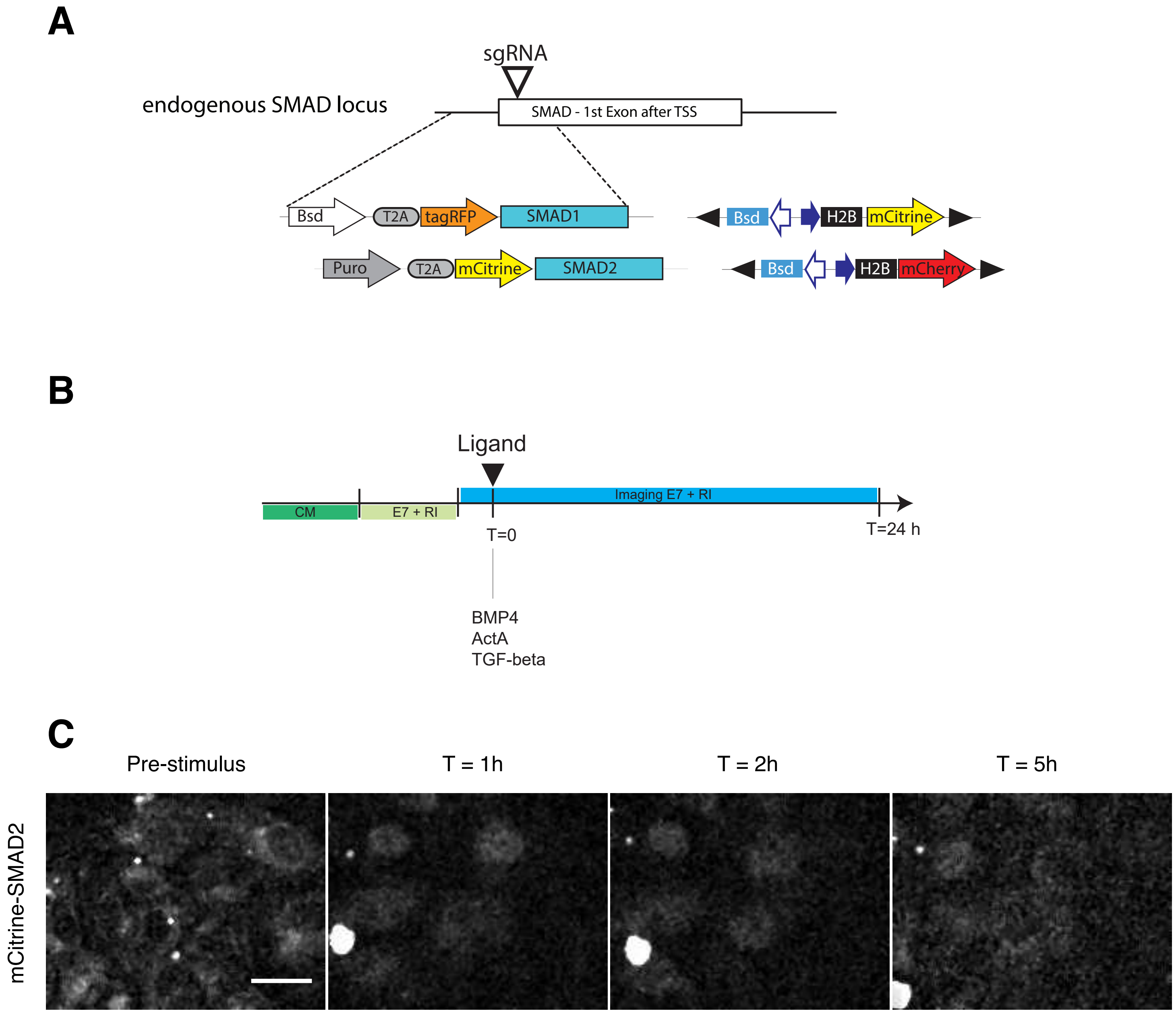
A
sgRNA
endogenous SMAD locus
SMAD - 1st Exon after TSS
Bsd
T2A
tagRFP
SMAD1
Bsd
H2B
mCitrine
Puro
T2A
mCitrine
SMAD2
Bsd
H2B
mCherry
B
Ligand
CM
E7 + RI
Imaging E7 + RI
T=0
T=24 h
BMP4
ActA
TGF-beta
C
Pre-stimulus
T = 1h
T = 2h
T = 5h
mCitrine-SMAD2

Fig. S4.

Generation and characterization of SMAD reporter cell lines in CAG-expanded genetic background.

A. Schematic diagram showing CRISPR-Cas9 mediated gene editing by targeting endogenous SMAD loci. SMAD1/2 fusion proteins were generated by tagging their N-terminal using tagRFP (for SMAD1) and mCitrine (for SMAD2) fluorophores. Each line was co-transfected with an ePiggyBac transposable element carrying H2B nuclear marker fused to a different fluorophore. This allowed us to track individual cells during the experiment and to quantify their nuclear SMAD signal as a response to the ligand.

B. Experimental setup in a defined TeSR-E7 medium devoid of TGFβ ligands. Cells were propagated in conditioned medium, dissociated into single cells and kept in E7+Rock inhibitor throughout the experiment. To reduce phototoxicity Imaging-E7 was used during data collection. Images were acquired every 10 mins for a duration of 24 hours following ligand presentation.

C. Snapshots capturing mCitrine-SMAD2 response over time in conditioned medium in 20CAG genetic background.

Scale bar: 50 µm.

Development • Supplementary information

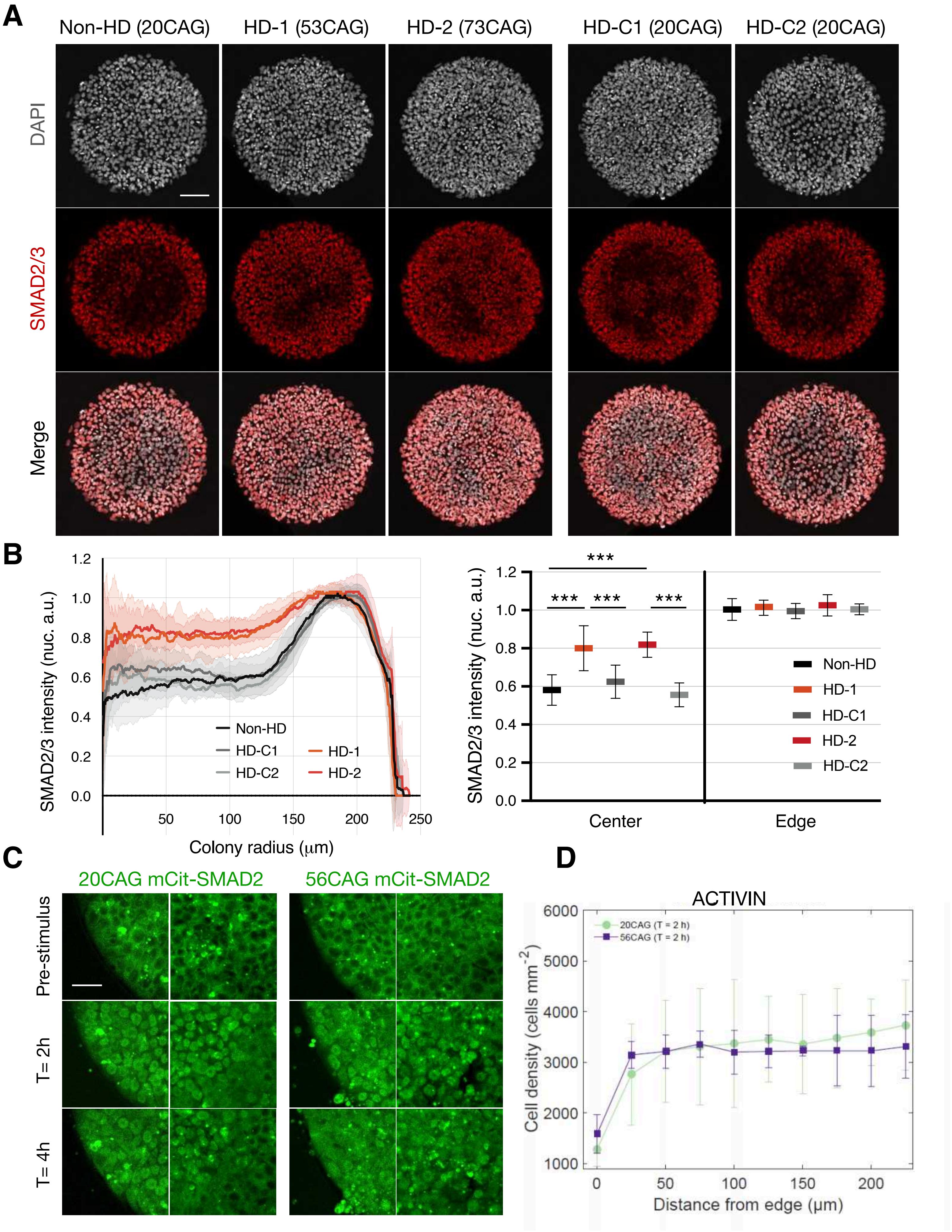

Development • Supplementary information

Fig. S5.

Characterization of spatial SMAD2/3 response in HD iPSCs and HD hESC mCit-SMAD2 live reporter.

A. Immunofluorescence of SMAD2/3 (red) in Non-HD and HD iPSCs (left) with their CRISPR-corrected counterparts (right) after 1 hour of ACTIVIN (100 ng/ml) stimulation in conditioned medium. DNA is visualized using DAPI (gray).

B. Mean radial intensity profile of nuclear SMAD2/3 based on immunofluorescence data of iPSCs plotted with s.d. of $n>15$ colonies (left). Mean radial intensity measures plotted in colony centers versus colony edges of 20CAGs (grays) and expanded CAG-length iPSCs (orange, red). ***$p<0.001$, Kruskal-Wallis followed by Dunn (right).

C. Snapshots of live cell imaging of 20CAG and 56CAG mCitrine-SMAD2 reporter cell lines in imaging quality TeSR-E7 medium lacking any TGFβ ligands. mCitrine-SMAD2 response to ACTIVIN (10 ng/ml) at the colony edge (left) and colony center (right) for both genotypes at pre-stimulus, 2 and 4 hours post stimulation.

D. Analysis of cell density at maximal nuclear to cytoplasmic mCitrine-SMAD2 signal in response to ACTIVIN (at T=2 h) in 20CAG and 56CAG.

Scale bars: 100 μm (a), 25 μm (c).

a.u. arbitrary units

Development • Supplementary information

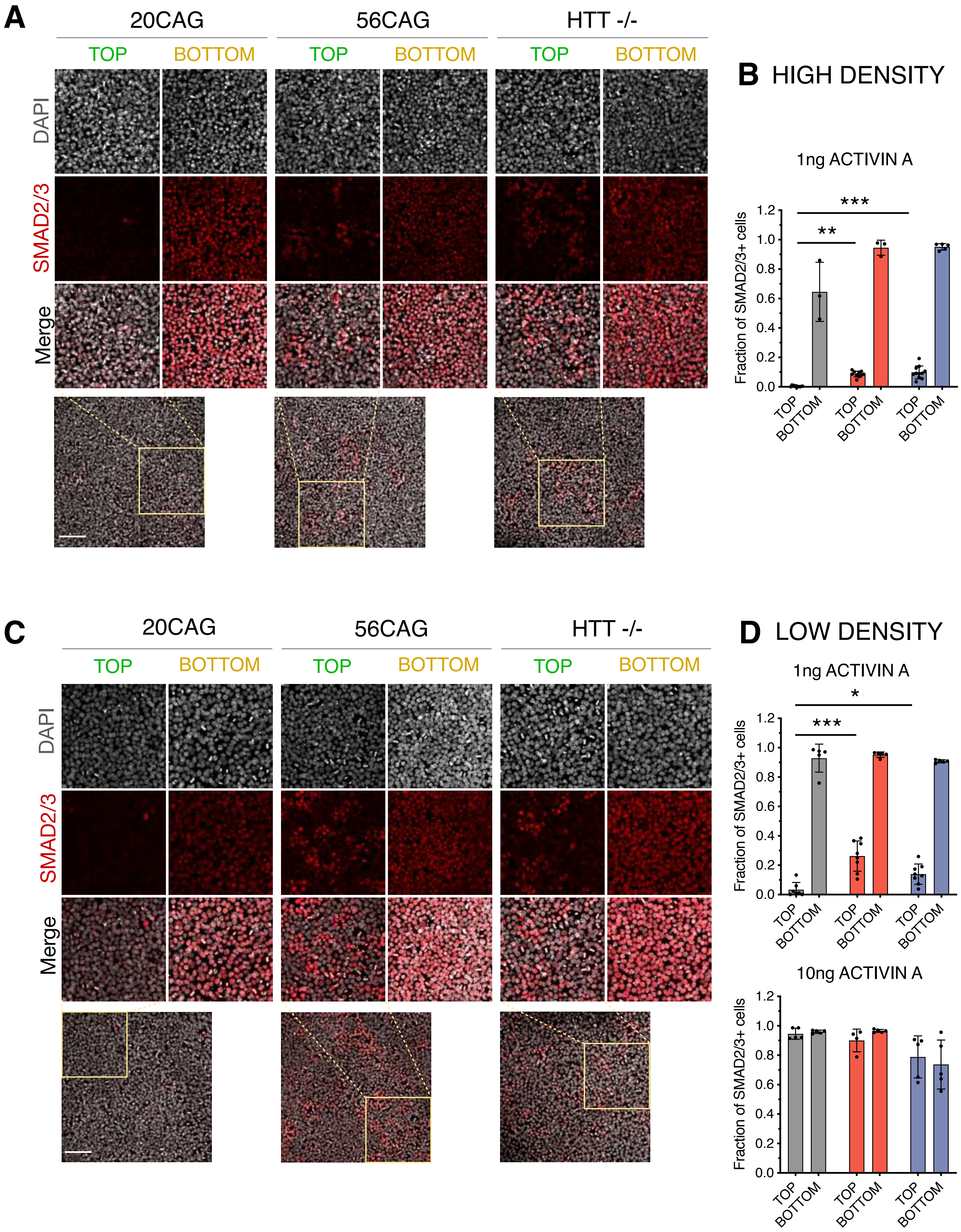

Development • Supplementary information

Fig. S6.

Analysis of functional ACTIVIN receptor localization of mutant hESC epithelia at different cell densities and ligand concentrations.

A. ACTIVIN receptor relocalization in high density hESC cultures assessed on transwell filters in TeSR-E6 medium 1 hour post stimulus. SMAD2/3 (red) activation probed by immunofluorescence and DNA visualized using DAPI (gray). ACTIVIN (1 ng/ml) presented from top or bottom compartment respectively.

B. Nuclear SMAD2/3 intensity in 56CAG and HTT $^{-/-}$ compared to 20CAG analyzed in individual cells. Fraction of activated cells are presented as mean ±s.d. after top and bottom ligand presentation. **$p<0.01$, ***$p<0.001$, ANOVA.

C. ACTIVIN receptor relocalization in low density hESC cultures assessed on transwell filters in TeSR-E6 medium 1 hour post-stimulus. SMAD2/3 (red) activation probed by immunofluorescence and DNA visualized using DAPI (gray) in 20CAG, 56CAG and HTT $^{-/-}$. ACTIVIN (1 ng/ml) presented from top or bottom compartment.

D. Nuclear SMAD2/3 intensity in 56CAG and HTT $^{-/-}$ compared to 20CAG analyzed at a single-cell resolution. Fraction of activated cells are presented as mean ±s.d. after top and bottom ligand presentation (top). At low density cells respond to 10 ng/ml ACTIVIN equally presented from top and bottom compartments independent of genotype (bottom). *$p<0.05$, ***$p<0.001$, ANOVA.

Scale bars: 100 μm.

Development • Supplementary information

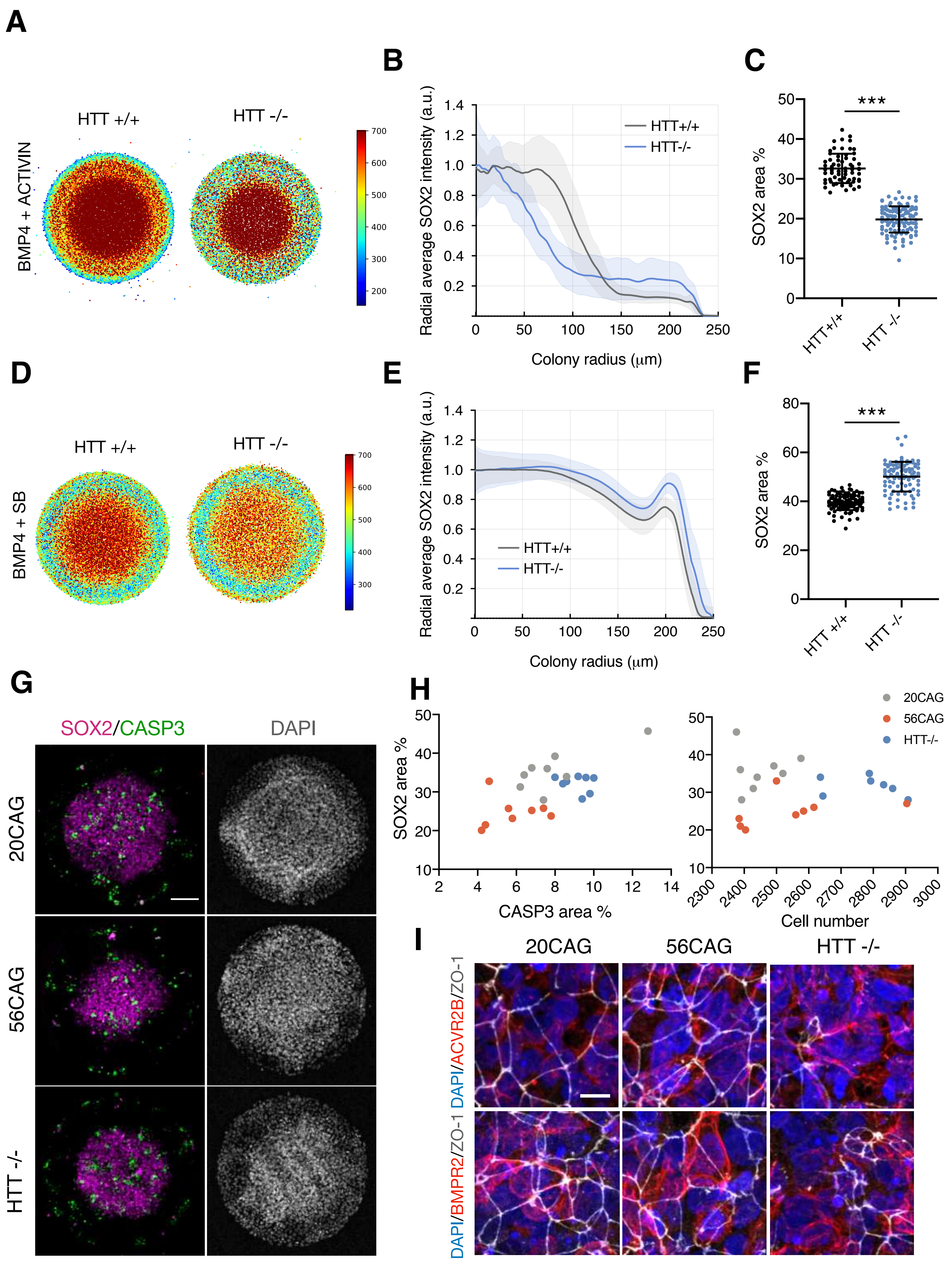
A
HTT +/+
HTT -/-
BMP4 + ACTIVIN
B
Radial average SOX2 intensity (a.u.)
HTT+/+
HTT-/-
Colony radius (μm)
C
SOX2 area %
***
HTT+/+
HTT -/-
D
HTT +/+
HTT -/-
BMP4 + SB
E
Radial average SOX2 intensity (a.u.)
HTT+/+
HTT-/-
Colony radius (μm)
F
SOX2 area %
***
HTT +/+
HTT -/-
G
SOX2/CASP3
DAPI
20CAG
56CAG
HTT -/-
H
SOX2 area %
CASP3 area %
Cell number
20CAG
56CAG
HTT-/-
I
20CAG
56CAG
HTT -/-
DAPI/ACVR2B/ZO-1
DAPI/BMPR2/ZO-1

Fig. S7.

Consequences of polarity defects in $HTT^{-/-}$ 2D gastruloids.

- **A.** Composite dot plots of relative SOX2 intensities in $HTT^{+/+}$ and $HTT^{-/-}$ 2D gastruloids following BMP4 (50 ng/ml) and ACTIVIN (100 ng/ml) differentiation for 48 hours in conditioned medium. Each dot corresponds to a single cell in the plots and composites of n>50 colonies in each genotype are presented (red=high SOX2 expression, blue=low SOX2 expression).
- **B.** Mean radial SOX2 intensity profile ±s.d. of n>10 colonies for each genotype in the BMP4+ACTIVIN condition. The plot was generated based on the immunofluorescence data.
- **C.** SOX2+ area was determined and plotted as the percentage of the total BMP4+ACTIVIN 2D gastruloid size (r=250 μm). Each dot represents a single colony in the scatter plot with mean ±s.d. of n>50 colonies obtained for each genotype. ***p<0.001, Mann Whitney.
- **D.** Relative SOX2 intensities displayed as composite dot plots of n>50 colonies in BMP4 (50 ng/ml) and SB431542 (10 μM) differentiated micropatterns. Single cell analysis was carried out in $HTT^{+/+}$ and $HTT^{-/-}$ at the 48 hours endpoint (red=high SOX2 expression, blue=low SOX2 expression).
- **E.** Mean radial SOX2 intensity profile ±s.d. of n>50 colonies for each genotype. The plot is based on the immunofluorescence data of BMP4+SB colonies.
- **F.** SOX2+ area was determined and plotted as the percentage of the total BMP4+SB 2D gastruloid size (r=250 μm). Each dot represents a single colony in the scatter plot with mean ±s.d. of n>50 colonies obtained for each genotype. ***p<0.001, Mann Whitney.
- **G.** SOX2 (magenta) and active CASPASE 3 (green) co-immunostaining of BMP4-induced micropatterns in 20CAG, 56CAG and HTT-/- at 48 hours endpoint. DNA stain DAPI is shown in gray.
- **H.** Quantitative analysis of SOX2+ area as a percentage of total 2D gastruloid size (r=250 μm) in the function of apoptotic activity measured by active CASPASE3 signal as a percentage of the colony size (left) or in the function of total cell number counted in the colonies (right). The measurements were performed for n=8 representative colonies based on immunofluorescence data in each genotype.
- **I.** Immunofluorescence data of endogenous ZO-1 (green) and transiently expressed exogenous TGFβ receptors: ACVR2B-HA (red, top) and BMPR2-HA (red, bottom) induced by doxycycline in 20CAG, 56CAG and $HTT^{-/-}$. DAPI (blue) is used as a DNA stain.

  Similar results were obtained in n=2-3 independent experiments.

  Scale bars: 100 μm (G), 10 μm (I).

  a.u. arbitrary units

Development • Supplementary information

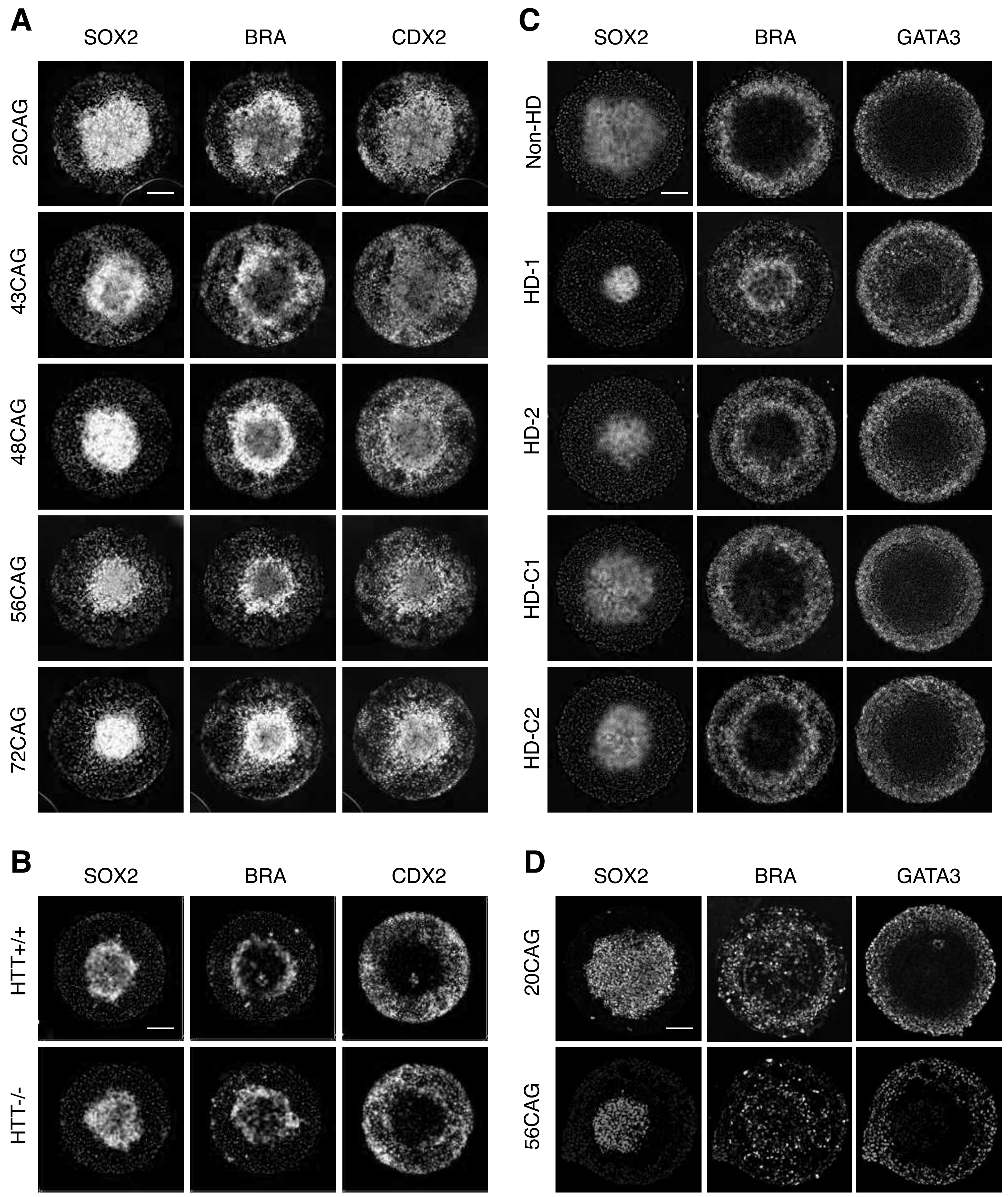

Fig. S8.

Grayscale images of 3-color gastruloids containing both red and green.

- **A.** Grayscale images corresponding to Figure 1B.
- **B.** Grayscale images corresponding to Figure 1G.
- **C.** Grayscale images corresponding to Figure S1C-D.
- **D.** Grayscale images corresponding to BMP4 condition of Figure 3B.

Scale bars: 100 µm.

Development • Supplementary information

Table S1. RT-qPCR primers

Gene symbol	Forward primer	Reverse primer
GAPDH	AATCCCATCACCATCTTCCA	TGGACTCCACGACGTACTCA
LEFTY1	CTCCATGCCGAACACCAG	GGAAAGAGGTTCAGCCAGAG
LEFTY2	TCAATGTACATCTCCTGGCG	CTGGACCTCAGGGACTATGG
CER1	GCCATGAAGTACATTGGGAGA	CACAGCCTTCGTGGGTTATAG
EOMES	CACATTGTAGTGGGCAGTGG	CGCCACCAAACTGAGATGAT
GSC	GAGGAGAAAGTGGAGGTCTGGTT	CTCTGATGAGGACCGCTTCTG
MIXL1	CCGAGTCCAGGATCCAGGTA	CTCTGACGCCGAGACTTGG

Table S2. Antibody information

Antigen	Antibody	Dilution
SOX2	Cell Signaling 3579S rabbit mAb	1:200
SOX2	Cell Signaling 4900S mouse mAb	1:100
BRACHYURY	R&D Systems AF2085 goat polyAb	1:300
BRACHYURY	R&D Systems MAB20851 rabbit mAb	1:200
CDX2	Abcam AB15258 mouse mAb	1:100
GATA3	Thermo Fisher Scientific MA1-028 mouse mAb	1:100
SOX17	R&D Systems AF1924 goat polyAb	1:200
SOX17	Abcam AB84990 mouse mAb	1:200
pSMAD1	Cell Signaling 9516S rabbit mAb	1:100
SMAD2/3	BD Biosciences 610842 mouse mAb	1:100
ZO-1	Thermo Fisher Scientific 61-7300 rabbit polyAb	1:200
HA	Millipore Sigma 11867423001 rat mAb	1:200
Active CASPASE 3	R&D Systems AF835 rabbit polyAb	1:500
