# Supplementary material for: Huntingtin CAG expansion impairs germ layer patterning in synthetic human 2D gastruloids through polarity defects
Source: Development. 2021 Oct 5;148(19):dev199513. doi: 10.1242/dev.199513 (PMC8513611; doi:10.1242/dev.199513)
Supplement: Reviewer comments [file develop-148-199513_review_history.pdf]

Development

Huntingtin CAG expansion impairs germ layer patterning in synthetic human 2D gastruloids through polarity defects.

Szilvia Galgoczi, Albert Ruzo, Christian Markopoulos, Anna Yoney, Tien Phan-Everson, Shu Li, Tomomi Haremaki, Jakob J. Metzger, Fred Etoc and Ali H Brivanlou

DOI: 10.1242/dev.199513

Editor: James Wells

Review timeline

Original submission:	5 February 2021
Editorial decision:	22 March 2021
First revision received:	1 July 2021
Accepted:	20 August 2021

Original submission

First decision letter

MS ID#: DEVELOP/2021/199513

MS TITLE: Huntingtin CAG expansion impairs germ layer patterning in synthetic human gastruloids through polarity defects.

AUTHORS: Szilvia Galgoczi, Albert Ruzo, Christian Markopoulos, Anna Yoney, Tien Phan-Everson, Tomomi Haremaki, Jakob J. Metzger, Fred Etoc, and Ali H Brivanlou

I have now received all the referees' reports on the above manuscript, and have reached a decision. The referees' comments are appended below, or you can access them online: please go to BenchPress and click on the 'Manuscripts with Decisions' queue in the Author Area.

As you will see, the referees express considerable interest in your work, but have some significant criticisms and recommend a substantial revision of your manuscript before we can consider publication. If you are able to revise the manuscript along the lines suggested, which may involve further experiments, I will be happy receive a revised version of the manuscript. Your revised paper will be re-reviewed by one or more of the original referees, and acceptance of your manuscript will depend on your addressing satisfactorily the reviewers' major concerns. Please also note that Development will normally permit only one round of major revision.

We are aware that you may be experiencing disruption to the normal running of your lab that make experimental revisions challenging. If it would be helpful, we encourage you to contact us to discuss your revision in greater detail. Please send us a point-by-point response indicating where you are able to address concerns raised (either experimentally or by changes to the text) and where you will not be able to do so within the normal timeframe of a revision. We will then provide further guidance. Please also note that we are happy to extend revision timeframes as necessary.

Please attend to all of the reviewers' comments and ensure that you clearly highlight all changes made in the revised manuscript. Please avoid using 'Tracked changes' in Word files as these are lost in PDF conversion. I should be grateful if you would also provide a point-by-point response detailing how you have dealt with the points raised by the reviewers in the 'Response to Reviewers' box. If you do not agree with any of their criticisms or suggestions please explain clearly why this is so.

© 2021. Published by The Company of Biologists under the terms of the Creative Commons Attribution License (https://creativecommons.org/licenses/by/4.0/).

Reviewer 1

Advance summary and potential significance to field

Galgoczi and colleagues present an interesting analysis of the effect of mutant huntingtin during early development, specifically during gastrulation. The author used a synthetic human 2D gastruloid model generated from an impressive number of isogenic ESCs of different CAG repeats of mutant HTT thereby reproducing the spectrum of the severity of HD. In doing so, the author described a CAG length-dependent reduction in the ectodermal compartment with concomitant expansion in the endodermal population that the authors attributed to disrupted TGFb signaling. The technical aspects of this study are impressive and its use to study the physiopathology of HD is not only novel but unique. Overall, this study has a sound rationale, methodology, and provides a 'developmental' view of disease that would benefit the field. However, there are few concerns that authors should address.

Comments for the author

Major comments:

1. The main assumption is that 2D gastruloid model reproduces human development. The author should discuss the limitations of such a model that is missing the multilayered complexity of a 3D embryo.

2. The rationale behind investigation of the TGFb signaling is based on previous observations by authors that Noggin induction and basolateral receptor localization are crucial for gastruloid pattern formation. There is a missed opportunity here to perform a more in-depth and unbiased analysis of gene expression profile (i.e. bulk or single cell RNA sequencing) that would yield a more comprehensive repertoire of HD associated changes during embryogenesis.

3. The full-length huntingtin/mutant huntingtin proteins have various functions in the cell and can be cleaved into smaller fragments. Nuclear aggregates are mainly regarded as toxic and being a hallmark of the of the pathology. Are there cytoplasmic or nuclear aggregates in this model that would indicate fragmentation?

4. HTT knockout mice die early in development, while mutant HTT homozygotes are viable. Thus, the mutant HTT must retain a level functionality that allows for "proper" development. Early alterations depicted during development, including during gastrulation, as in the present work, suggest the presence of compensatory mechanisms that fully rescue the disease phenotypes. This is also likely the case in human development. Presymptomatic carrier patients have normal motor and intellectual capabilities (e.g. Nancy Wexler, a lead figure in HD research and Sarah Winckless, an Olympic medalist). Therefore, the relevance of mutant HTT-associated alterations during early development must be neutralized by compensatory mechanisms that have yet to be identified. While such compensatory mechanisms are beyond the scope of this manuscript, the author should nonetheless alert the reader that such mechanisms exist and that the developmental hypothesis of HD does not alone warrant preventive treatments at the prodromal stage as suggested in the conclusion.

5. The authors used Gapdh to normalize the Q-PCR data. Gapdh expression is known to be altered in certain disease states. Do Gapdh levels change in these cells?

Minor comments:
1. The reference #10 describe a loss of function (not a gain of function) and more specifically a reduced BDNF signaling in HD.

2. The following sentence is incomplete:
"Moreover, HTT-/- phenocopies results obtained from CAG-expanded lines in a self-organizing model of human neurula (neuruloids), and hESC-derived neurons."

© 2021. Published by The Company of Biologists under the terms of the Creative Commons Attribution License (https://creativecommons.org/licenses/by/4.0/).

3. The second sentence in the discussion is not clear. The association between the prodromal phase in HD (the phase when early symptoms are present) and embryogenesis is not clear.

4. HD is an aging disorder in humans but in its severe form, the disease has a juvenile-onset. This is not a hallmark of neurodevelopmental disorder as the author suggested but rather of an augmented toxicity of the mutant HTT.

Reviewer 2

Advance summary and potential significance to field

In this manuscript, the authors use 2D “gastruloids” - geometrically confined micropatterned cultures of hPSCs - to demonstrate that HTT mutations, both CAG expansion repeats (CAG-HTT; causal to Huntingon’s Disease) and HTT-KO, cause severe disruption of the self-organized concentric circles representing the germ layers. I thank the authors for sending the Nature Comm reviews and rebuttal, and can appreciate how much important new experiments have been performed while the paper was under review there. This version of the manuscript is well-written, the data are clearly presented (although I have some minor comments on this, see below) and the findings are potentially of broad scientific interest. However, the authors try to draw straightforward conclusions from their experiments. This is not fully justified by the data and in light of some literature the authors do not cite (PMID: 23967334). Also, in light of the author’s findings, it will certainly be of interest to cite & discuss & PMID: 29311338.

Below, I will detail critical points that should be experimentally addressed and some suggestions that could strengthen the paper. In addition, I have some minor comments.

Comments for the author

Critical points:
In general, an important paper that the authors do not cite is PMID: 23967334 (Functions of Huntingtin in Germ Layer Specification and Organogenesis by Nguyen & al). In that paper, the effects of HTT KO & CAG-HTT (Q111 allele) on germ layer specification in (a.o.) Embryoid Bodies (EBs) is assessed. As the authors also describe in the submitted manuscript, there are remarkable differences between HTT KO & CAG-HTT. Overall, PMID: 23967334 demonstrates essential roles of HTT in the specification of the three germ layers and cell survival and, importantly, that these developmental events are differentially deregulated by CAG-HTT (Q111). More specifically, PMID: 23967334 describes how:
cell death is specifically and significantly increased in HTT-KO EBs - appears to be more pronounced in non-neural lineages HTT regulates the specification, maintenance and survival of mesendodermal progenitors HTT is required for the specification of mesendodermal progenitors and survival of neuroectodermal progenitors, whereas CAG-HTT (Q111) promotes precocious specification of the neuroectodermal fate.
Nodal is upregulated in both HTT-KO & CAG-HTT EBs

Thus, MAYBE, HTT-KO leads to impaired survival of mesendoderm progenitors in 2D gastruloids, and this is overcome by ACTIVIN addition; thereby explaining the discrepancy between the CAG-HTT and HTT-KO phenotype that is overcome by ACTIVIN supplementation. Moreover, the observations of PMID: 23967334 leave the possibility that also in 2D gastruloids precocious differentiation of ectodermal cells occurs. In fact, some of the data the authors show appear to back this possibility (as detailed below). What would happen to these cells? Certainly they would loose the pluripotency & early neural marker SOX2, but could they transdifferentiate when confronted with the appropriate cues?

In any case, the authors should cite & discuss their data in light of the findings of PMID: 23967334. More specific comments, some of which follow from the short discussion above:

The authors only provide quantitative information on the Sox2 domain (intensity/radius). These analyses should be complemented with analysis of the other domains, given that HTT has been shown to also be involved in the specification of other germ layers

© 2021. Published by The Company of Biologists under the terms of the Creative Commons Attribution License (https://creativecommons.org/licenses/by/4.0/).

(PMID: 23967334). Proper quantification would also help to substantiate the claim that the differentiation potential of the hPSCs is not affected. Yes, all germ layers can be formed, but what is the (relative) propensity - besides the clear effect on ectoderm formation?

Similarly, the total number of cells of each germ layer should be quantified, as well as the total number of cells in the micropattern. E.g. in Fig 1c it seems that especially in the 72CAG condition the total number of cells is much lower. Is this indeed the case and if so, what is the reason for this? Is it increased apoptosis? Decreased proliferation (possibly due to precocious differentiation? Decreased cell density could impact e.g. germ layer formation via quorum-sensing impact forces (especially given that in micropatterns boundaries are fixed). I understand that it is difficult and beyond the scope of the manuscript to dissect all these possible scenarios, but the authors should at least provide a time-resolved (obtained by fixation and IFC at multiple timepoints) analysis of cell density, both for total number of cells (DAPI) and germ layer specific markers.
This is especially relevant, since, as the authors acknowledge themselves in the manuscript, receptor re-localization in the gastruloids is density-dependent.

Related to 2, a similar time-resolved analysis of apoptosis should be performed across the CAG conditions and in HTT-KO.

Fig 1i: the data indicate increased activation of Sox2 at 20h in 56CAG and 72CAG compared to 20CAG. The authors should quantify this (at present, only a quantification of the radius is provided, but this should be complemented with the time-resolved quantification of the signal intensity and the absolute number of cells). This is especially important, given the possibility of precocious specification of the neuroectodermal fate. Providing this analysis is also important to substantiate the claim of the authors that “This demonstrates that HTT mutation does not modify the early cell-intrinsic response to BMP4”, which is currently not backed by any quantitative data.
In fact, it would be very helpful if the authors would analyze the expression of other (early) neural markers, e.g. Sox1, Pax6 to assess the possibility of precocious neuroectodermal differentiation. Sox2 is not very informative, since it is also a pluripotency marker and thus expressed from start.
Related to point 4) and 5): whereas it’s true that at the culture endpoint there’s no obvious difference between HTT+/+ and HTT-/- (Fig 1f,g), the dynamics of reaching this endpoint might be very different. This could be studied by generating HTT-/- cells with the Sox2-mCit construct as done for the CAG-HTT (Fig 1h-j).

The epistasis experiments are important, but very difficult to interpret with the current presentation of the data. All relevant conditions should be presented in the same panel (e.g. those presented in Fig. S3a, c, d; for instance CHIR only).

Also, some critical conditions are missing. What is the effect of WNT3a alone? Of Activin alone? Especially the latter is of critical importance, given the effect of CHIR + Activin in HTT-/-. Also, interpretation of these experiments is difficult given the obvious effects of small molecules on germ layer specification in general (independent of the HTT mutations) and may impact cell behaviour (proliferation, apoptosis). If certain germ layers are not specified, this will also disrupt the endogenous signals secreted from these germ layers. Also certainly cells just fill up the space available, even if certain germ layers are not formed? E.g. in BMP4 + IWP2 a similar number of SOX2+ cells may spread over a bigger area in the absence of a mesodermal BRA+ ring. It follows that not only relative ratios of the domains, but also ratios of absolute cell numbers of the different germ layers (and possibly intensities, see point 1 & 4) should be provided.
I don’t understand the conclusion of the epistasis experiments that “an influence of WNT signaling cannot completely be excluded”. In fact, IWP2 leads to a 13% reduction of the center. Moreover, CHIR + Activin leads to a 60% reduction, and the effect of Activin alone has not been addressed (see point 6).

The conclusion that “HTT-CAG expansion affects cell-intrinsic signaling without affecting the transcriptional output… must arise from a non-cell autonomous effect” is based on the analysis of only a few marker genes. Certainly the possibility that cells transdifferentiate can not be excluded? Also, I fail to see how this experiment - while elegant! - can reliably make predictions about cell-intrinsic vs non-cell autonomous effects of cells on micropatterns, where cell communication may lead to all kinds of differences in molecular and cellular processes.

© 2021. Published by The Company of Biologists under the terms of the Creative Commons Attribution License (https://creativecommons.org/licenses/by/4.0/).

The authors should clarify the reason for using different concentrations of ACTIVIN & BMP4 in the micropattern (50 resp 100 ng/ml) vs the transwell (10 resp 10 ng/ml) assays? What are the effects of these in WT 2D gastruloids? How could this impact the effects of HTT-CAG and HTT-/-? Are the same effects in the transwell assays observed when using the concentrations of the micropattern assay and vice versa?
Other suggestions:
The analysis are all based on the analysis of a very limited set of marker genes.
The authors could expand with e.g. IFC of early neural markers to test for precocious differentiation and/or transdifferentiation using the Sox2-mCitrine line. Alternatively, with the same line cells could be FACS-purified at different timepoints between 0-40h to analyze transcriptomic changes possibly indicative of precocious and/or transdifferentiation.
The authors state that "we cannot fully exclude that signal propagation is also shaped by secreted inhibitors". The authors could perform immunolocalization or ISH to test this. Same for the localization of BMP receptors (as mentioned in the next sentence).
As described in (a.o.) PMID: 23967334 both CAG-HTT & HTT-KO could upregulate Nodal. Could the authors analyze if this is also the case in their system? If so how would this affect the patterning of the 2D gastruloids, given the important role of Nodal in this process?
Adding a ubiquitous nuclear reporter to the Sox2-mCit line would provide a tool to assess the fate of the Sox2-mCit+ cells.

Minor comments:
Although the authors are right that 2D gastruloids are an excellent platform for obtaining quantitative insights, it should be made unequivocally clear that this is a 2D model, not to be confused with 3D gastruloids. The authors should replace "gastruloids" throughout the paper with "2D gastruloids". I also suggest that the authors mention 3D gastruloids (Moris et al., 2020) and acknowledge the limitations of a 2D system to study a 3D process in the discussion of their manuscript.

Throughout the figures, color-blind friendly color combinations should be used. In particular the authors should refrain from using red-green combinations (instead used magenta-green or red-blue). In cases where this is not possible (e.g. 3-color combinations), please provide the separate channels in grayscale in a supplemental figure in addition to the merged panel in the main figure.

Although the manuscript is in general clearly and well-written, there are multiple grammar and spelling errors throughout the manuscript. Please carefully reassess the text to correct these.
The sample sizes are not consequently provided in all of the figure legends (e.g. Fig 1B). Please ensure that this is the case where relevant. In particular, sample sizes are not provided in the imaging panels. How many times was the same (or similar) result achieved? Please add an n/N to the figures ("the shown phenotypes was observed in n colonies out of N colonies in X independent experiments").

The resolution of some of the imaging data is very low (e.g. Fig 1C). Please provide higher resolution data.

Could the authors explain why in Fig 1E there's a clear effect in the 43CAG and 48CAG condition whereas this is supposed to represent late onset Huntington's Disease?

Fig. 2f: Are these values normalized for DAPI intensity? It's (very) hard to appreciate the quantified differences based on the stainings in Fig. 1d.

Fig. 4a: where are the raw data of the other conditions (of which a quantification is provided in Fig 4b)?

In Fig 7a & 7c there are mysterious colored rectangles on top of the figure where most likely the coloring legend of the staining should be presented. Same for Fig 6a on the left side.

How physiologically relevant is the observed expansion of the endodermal population? Are there any abnormalities in endoderm-derived associated with Huntington's disease?

© 2021. Published by The Company of Biologists under the terms of the Creative Commons Attribution License (https://creativecommons.org/licenses/by/4.0/).

“Impaired spatial restriction to ACTIVIN signaling observed in HTT-CAG expanded colonies suggest a failure of this mechanism…”. Do cells first transdifferentiate (if they do?) before or after spatial restriction of ACTIVIN is impaired? In other words, cause or consequence?

I don’t understand why “in HTT-/- line ZO-1 expression was selectively absent in cells responding to apical ACTIVIN stimulation”. Would tight junction integrity not lead to ACTIVIN freely diffusing to the apical side by impairing barrier function, thus also affecting other cells?

“In human gastruloids, this led to enhanced mesendodermal differentiation and as a consequence a smaller ectodermal domain”. In the absence of live tracking of cells labelled with mesendodermal and ectodermal markers there’s no unequivocal evidence that this is the cause-consequence sequence.

“Since the 56CAG extended line phenocopies…this observation confirms that cellular polarization is compromised in the HTT-CAG expanded background, even though expression of ZO-1 remains intact.” There could be other explanations, e.g. different developmental trajectories for HTT-CAG and HTT-/- to reach the same endpoint, with compromised polarization being important for HTT-/- phenotype, but ACTIVIN triggering transdifferentiation potential in the HTT-CAG line.
The authors should remove the last half-sentence “could shift the clinical focus….during the prodromal phase”. This is unnecessary and speculative at best.

If the authors want to keep the sentence they should provide a clear example of how such prodromal clinical intervention could look like.

Fig S1c: why is the effect of 53CAG more severe than 73CAG?

Fig S1f: HD-C1 and HD-C2 vs Non-HD also seem significant? Please provide stats for those comparisons. If difference is significant, what could be the reason for that?

First revision

Author response to reviewers' comments

In this revised version we have addressed every point raised by both referees, which we believe has substantially improved the quality of our study. Following their advice, we have now performed a significant number of additional experiments and modified our manuscript to reflect the changes. These include: (i) 10 completely new panels to our main and supplementary Figures; (ii) new data to 4 existing panels; and (iii) a new Supplementary Figure 8 with grayscale images to accommodate color blindness (Reviewer #2). We also present a point-by-point response to each reviewer’s specific points.

We would like to thank both reviewers for their constructive criticisms and suggestions. Their comments have undoubtedly elevated the quality of our studies on the origin of Huntington’s Disease, in human models during a period of embryonic development that would otherwise be impossible to study. We hope that you and the reviewers will find this revised version of our manuscript acceptable for publication in Development.

Rebuttal Development, Szilvia Galgoczi et al.

REVIEWER COMMENTS
We would like to thank the reviewers for their constructive criticisms and suggestions which have substantially helped us improve our paper. Taking the reviewers’ recommendations under consideration, we have added 2 new panels in the main figures, 12 new supplementary figure panels and new data to 4 additional panels. Here, we present a point-by-point response to each reviewer’s specific points.

Reviewer #1
We would like to thank the reviewer for her/his compliments on our work: ***“The technical aspects of***

© 2021. Published by The Company of Biologists under the terms of the Creative Commons Attribution License (https://creativecommons.org/licenses/by/4.0/).

this study are impressive and its use to study the physiopathology of HD is not only novel but unique. Overall, this study has a sound rationale, methodology, and provides a 'developmental' view of disease that would benefit the field"

The reviewer, has also made both major and minor suggestions that we address point by point below:

Major comments:

1. "The main assumption is that 2D gastruloid model reproduces human development. The author should discuss the limitations of such a model that is missing the multilayered complexity of a 3D embryo."

Following the advice, we have now added the limitation of our 2D gastruloid model in the Discussion (Pages 16- 17).

"2. The rationale behind investigation of the TGFb signaling is based on previous observations by authors that Noggin induction and basolateral receptor localization are crucial for gastruloid pattern formation. There is a missed opportunity here to perform a more in-depth and unbiased analysis of gene expression profile (i.e. bulk or single cell RNA sequencing) that would yield a more comprehensive repertoire of HD associated changes during embryogenesis."

We agree with the reviewer. In fact, we have performed comparative single cell RNA-sequencing analysis to investigate additional aspects of HD-associated changes on a single cell level. Some of these results are attached below in response to specific inquiries of point #5 of this reviewer and a comment by Reviewer #2. The entire sets of results and analysis will be submitted in a follow up publication. With 7 main Figures, and 8 Supplementary Figures (for a total of 85 panels), we simply ran out of room.

"3. The full-length huntingtin/mutant huntingtin proteins have various functions in the cell and can be cleaved into smaller fragments. Nuclear aggregates are mainly regarded as toxic and being a hallmark of the of the pathology. Are there cytoplasmic or nuclear aggregates in this model that would indicate fragmentation?"

We thank the reviewer for raising this point. We have previously shown that HTT protein, in cells grown under either pluripotency and differentiation conditions, is exclusively localized to the cytoplasm and excluded from the nucleus (Ruzo et. al, 2018). High resolution microscopy has also confirmed in the same study, that cytoplasmic HTT localization is homogeneous without apparent aggregation. Our results are in agreement with the recently published study that shows lack of nuclear staining, apical localization of HTT, and absence of expression in the nuclei of HD human fetuses (Barnat et al., 2020). We therefore excluded the implication of toxic aggregates in this study.

"4. … the present work, suggest the presence of compensatory mechanisms that fully rescue the disease phenotypes… This is also likely the case in human development. Presymptomatic carrier patients have normal motor and intellectual capabilities (e.g. Nancy Wexler, a lead figure in HD research and Sarah Winckless, an Olympic medalist). Therefore, the relevance of mutant HTT- associated alterations during early development must be neutralized by compensatory mechanisms that have yet to be identified. <u>While such compensatory mechanisms are beyond the scope of this manuscript, the author should nonetheless alert the reader that such mechanisms exist and that the developmental hypothesis of HD does not alone warrant preventive treatments at the prodromal stage as suggested in the conclusion."</u>

We agree with the reviewer that compensatory mechanisms are very likely to be at play in the embryonic context to correct for the cellular defects that can be observed at many levels when exploring the impact of mutant HTT in cellular systems *in vitro*. While we do not understand how, nor demonstrate that developmental defects lead to clinical manifestation - as also pointed out by the reviewer - it is important to acknowledge that the so-called "developmental hypothesis" calls for intervention that can either fix potential underlying disease predispositions that are already in place before symptoms onset, whether they involve cellular consequences of developmental stages or homeostasis in the adult, or perhaps even sustain compensatory mechanisms potentially delaying

© 2021. Published by The Company of Biologists under the terms of the Creative Commons Attribution License (https://creativecommons.org/licenses/by/4.0/).

clinical onset. We have now added this to the discussion section of our manuscript (Page 19).

"5. The authors used Gapdh to normalize the Q-PCR data. Gapdh expression is known to be altered in certain disease states. Do Gapdh levels change in these cells?"

We thank the reviewer for raising this important point. We have previously reported that there are no changes in GAPDH expression during the early neurogenesis in our isogenic HD-hESCs (Haremaki et al., 2018). Moreover, our single cell RNA-seq data (mentioned in response to point #2) also demonstrates that expression of GAPDH is not altered by the HD mutation in any of the germ layers following BMP stimulation in our cells.

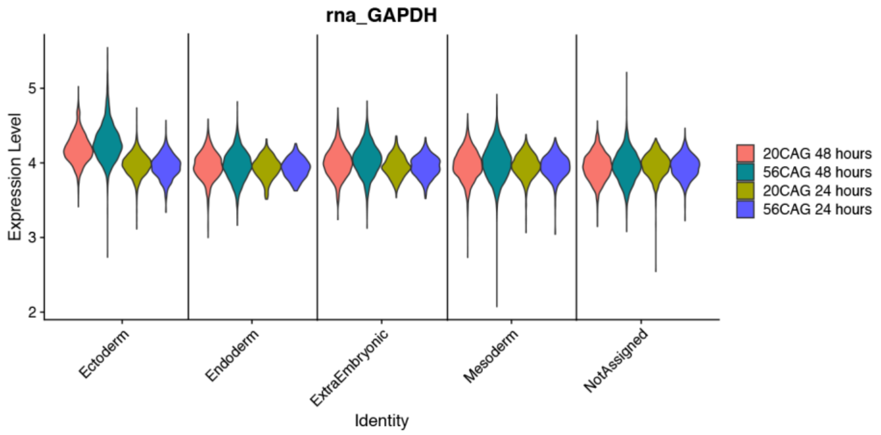

Figure 1: RNA expression of GAPDH in different germ layer populations obtained from single-cell RNA sequencing data set.

Minor comments:

"1. The reference #10 describe a loss of function (not a gain of function) and more specifically a reduced BDNF signaling in HD."

We corrected the mistake, we thank the reviewer for pointing it out.

"2. The following sentence is incomplete:
"Moreover, HTT-/- phenocopies results obtained from CAG-expanded lines in a self-organizing model of human neurula (neuruloids), and hESC-derived neurons."

The sentence has been corrected, we thank the reviewer for pointing it out.

"3. The second sentence in the discussion is not clear. The association between the prodromal phase in HD (the phase when early symptoms are present) and embryogenesis is not clear."

The sentence has been clarified, to remove the association between the prodromal phase of HD and embryogenesis. We thank the reviewer for pointing it out.

"4. HD is an aging disorder in humans but in its severe form, the disease has a juvenile-onset. This is not a hallmark of neurodevelopmental disorder as the author suggested but rather of an augmented toxicity of the mutant HTT."

We recognize that aging plays a crucial role in HD pathogenesis, but we find it important to acknowledge that it cannot fully explain the juvenile form of the disease. With that in mind, we clarified the sentence pointed out by the reviewer.

We would like to thank Reviewer #1 again for the constructive criticism that has undoubtedly elevated the quality of our manuscript.

© 2021. Published by The Company of Biologists under the terms of the Creative Commons Attribution License (https://creativecommons.org/licenses/by/4.0/).

Reviewer #2
We appreciate very much the reviewer's positive comments: ***"I thank the authors for sending the Nature Comm reviews and rebuttal, and can appreciate how much important new experiments have been performed while the paper was under review there. This version of the manuscript is well-written, the data are clearly presented (although I have some minor comments on this, see below) and the findings are potentially of broad scientific interest."***

The reviewer has also provided constructive criticism and suggestions that we address point-by-point below:

Reviewer 2 Comments for the Author:

Critical points:

"In general, an important paper that the authors do not cite is PMID: 23967334 (Functions of Huntingtin in Germ Layer Specification and Organogenesis by Nguyen & al). In that paper, the effects of HTT KO & CAG-HTT (Q111 allele) on germ layer specification in (a.o.) Embryoid Bodies (EBs) is assessed. As the authors also describe in the submitted manuscript, there are remarkable differences between HTT KO & CAG-HTT. Overall, PMID: 23967334 demonstrates essential roles of HTT in the specification of the three germ layers and cell survival and, importantly, that these developmental events are differentially deregulated by CAG-HTT (Q111). More specifically,
PMID: 23967334 describes how:

Cell death is specifically and significantly increased in HTT-KO EBs - appears to be more pronounced in non-neural lineages HTT regulates the specification, maintenance and survival of mesendodermal progenitors HTT is required for the specification of mesendodermal progenitors and survival of neuroectodermal progenitors, whereas CAG-HTT (Q111) promotes precocious specification of the neuroectodermal fate. Nodal is upregulated in both HTT-KO & CAG-HTT EBs. Thus, MAYBE, HTT-KO leads to impaired survival of mesendoderm progenitors in 2D gastruloids, and this is overcome by ACTIVIN addition; thereby explaining the discrepancy between the CAG- HTT and HTT-KO phenotype that is overcome by ACTIVIN supplementation. Moreover, the observations of PMID: 23967334 leave the possibility that also in 2D gastruloids precocious differentiation of ectodermal cells occurs. In fact, some of the data the authors show appear to back this possibility (as detailed below). What would happen to these cells? Certainly, they would lose the pluripotency & early neural marker SOX2, but could they transdifferentiate when confronted with the appropriate cues?

In any case, the authors should cite & discuss their data in light of the findings of PMID: 23967334."

We thank the reviewer for pointing to our omission. We have now cited and discussed PMID: 23967334 and modified the manuscript to reflect on the three main points (Pages 18-19). First, in regard to cell death, we have now systematically compared total cell numbers and tested for apoptosis in 20CAG, 56CAG and HTT-/- gastruloids (Figure S7g-h). As also discussed in point #3, we did not detect decreased cell density or enhanced apoptosis in 56CAG or HTT-/- compared to 20CAG. Therefore, we exclude the possibility that HTT-/- significantly compromises the survival of ectodermal or mesendodermal lineages in the context of gastruloid formation of these cell lines. Second, regarding precocious differentiation, we could not find evidence that 56CAG is further down the neuroectodermal trajectory than 20CAG at the endpoint of our assay. Please also see point #5 and Figure 2 below. Finally, regarding trans-differentiation, we believe it to be very unlikely in our system because: (i) the exit from pluripotency can be timed for t=20-25 hours in both non-HD and HD cell lines (as demonstrated by the SOX2-mCitrine dynamics), leaving a very short window of time for cells to commit to a specific germ layer and then transdifferentiate; (ii) in none of our own previously published 2D gastruloid work, or studies published by other groups, no sign of SOX2+/BRA+ bi-progenitors or trans-differentiation has been detected and germ layer specification has been solely attributed to the wave of signaling induced by the presented ligand (Warmlflash et al., 2015, Martyn et al., 2018, Minn et al., 2020); (iii) an enhanced signaling response in the HD cell lines has been detected as early as t=1 hour in pluripotency. Taken together, while we cannot completely exclude the

© 2021. Published by The Company of Biologists under the terms of the Creative Commons Attribution License (https://creativecommons.org/licenses/by/4.0/).

possibility that HTT-CAG expansion induces trans-differentiation, our results strongly support that the enhanced mesendodermal differentiation is due to enhanced signal reception in HD-RUES2.

More specific comments, some of which follow from the short discussion above:

"1. The authors only provide quantitative information on the Sox2 domain (intensity/radius). These analyses should be complemented with analysis of the other domains, given that HTT has been shown to also be involved in the specification of other germ layers (PMID: 23967334). Proper quantification would also help to substantiate the claim that the differentiation potential of the hPSCs is not affected. Yes, all germ layers can be formed, but what is the (relative) propensity - besides the clear effect on ectoderm formation?"

We agree with the reviewer and we have now quantified the percentage of positive cells in each of the germ layers in 20CAG, 43CAG, 48CAG, 56CAG and 72CAG presented in the new Figure S1g. Additionally, we now provide single cell quantification of the mesodermal and endodermal domains that was presented in Figure 3, in new panels of Figure S3f. This complements the quantification of our isogenic collection that we had previously published (Ruzo et. al, 2018).

"2. Similarly, the total number of cells of each germ layer should be quantified, as well as the total number of cells in the micropattern. E.g. in Fig 1c it seems that especially in the 72CAG condition the total number of cells is much lower. Is this indeed the case and if so, what is the reason for this? Is it increased apoptosis? Decreased proliferation (possibly due to precocious differentiation?
Decreased cell density could impact e.g. germ layer formation via quorum-sensing, impact forces (especially given that in micropatterns boundaries are fixed). I understand that it is difficult and beyond the scope of the manuscript to dissect all these possible scenarios, but the authors should at least provide a time- resolved (obtained by fixation and IFC at multiple timepoints) analysis of cell density, both for total number of cells (DAPI) and germ layer specific markers. This is especially relevant, since, as the authors acknowledge themselves in the manuscript, receptor re-localization in the gastruloids is density-dependent."

We would like to respectfully bring to the reviewer's attention that cell numbers have been quantified and are presented in Figure S2a. Nevertheless, we now provide additional quantification of cell density at early and at the endpoint of our assay at 48 hours following BMP4 stimulation. This data is presented in new Figures S2e and S7h, with cell number information for each domain in Figure S1g and S3f. Taken together, we excluded the possibility that the HD phenotypic signature is dictated by density differences.

"3. Related to 2, a similar time-resolved analysis of apoptosis should be performed across the CAG conditions and in HTT-KO."

Although, we did not detect enhanced cell death in the HTT-CAG expanded or HTT-/- gastruloids at any of the analyzed timepoints (1 hour, 24 hours and 48 hours), to address the reviewer's point, we tested for apoptosis by investigating the levels of active caspase 3, and cell number relative to SOX2 domain size in 20CAG, 56CAG, and HTT-/- after 48hours of stimulation. These results are presented in new panels of Figure S7g-h. We did not detect enhanced apoptosis in HTT-/- or 56CAG compared to 20CAG. Moreover, reduced SOX2 domain area did not correlate with enhanced apoptosis or reduced cell number. These new results are discussed on Pages 15 and 19.

"4. Fig 1i: the data indicate increased activation of Sox2 at 20h in 56CAG and 72CAG compared to 20CAG. The authors should quantify this (at present, only a quantification of the radius is provided, but this should be complemented with the time-resolved quantification of the signal intensity and the absolute number of cells). This is especially important, given the possibility of precocious specification of the neuroectodermal fate. Providing this analysis is also important to substantiate the claim of the authors that "This demonstrates that HTT mutation does not modify the early cell- intrinsic response to BMP4", which is currently not backed by any quantitative data."

We would like to respectfully bring to the reviewer's attention that the analysis of BMP4 response (at 1 hour and 24 hours post-stimulation) is presented in Figure 2 with information about cell density at

© 2021. Published by The Company of Biologists under the terms of the Creative Commons Attribution License (https://creativecommons.org/licenses/by/4.0/).

the 24 hour timepoint in Figure S2a. Nevertheless, we have now complemented with cell density analysis for the 1 hour timepoint in a new panel in Figure S2e. Figure 2 substantiates that the early pSMAD1 response of pluripotent cells is not altered by the HTT mutation, but the enhanced signaling response correlates with the timing of pluripotency exit at 24 hours confirming the observations in Figure 1i-j. Thus, the cell number analysis at multiple timepoints confirms that our results are not artefacts of density differences. (Also see the answer to the point below).

"5. In fact, it would be very helpful if the authors would analyze the expression of other (early) neural markers, e.g. Sox1, Pax6 to assess the possibility of precocious neuroectodermal differentiation. Sox2 is not very informative, since it is also a pluripotency marker and thus expressed from start."

We agree with the reviewer. In our currently unpublished single cell RNA-seq (discussed in point #2 and #5 of Reviewer #1) presented in Figure 2 below, we did not detect SOX1 or PAX6 expression in 20CAG nor 56CAG during our differentiation window. Based on these results 56CAG does not appear to be more downstream the neuroectodermal trajectory than 20CAG at this stage.

© 2021. Published by The Company of Biologists under the terms of the Creative Commons Attribution License (https://creativecommons.org/licenses/by/4.0/).

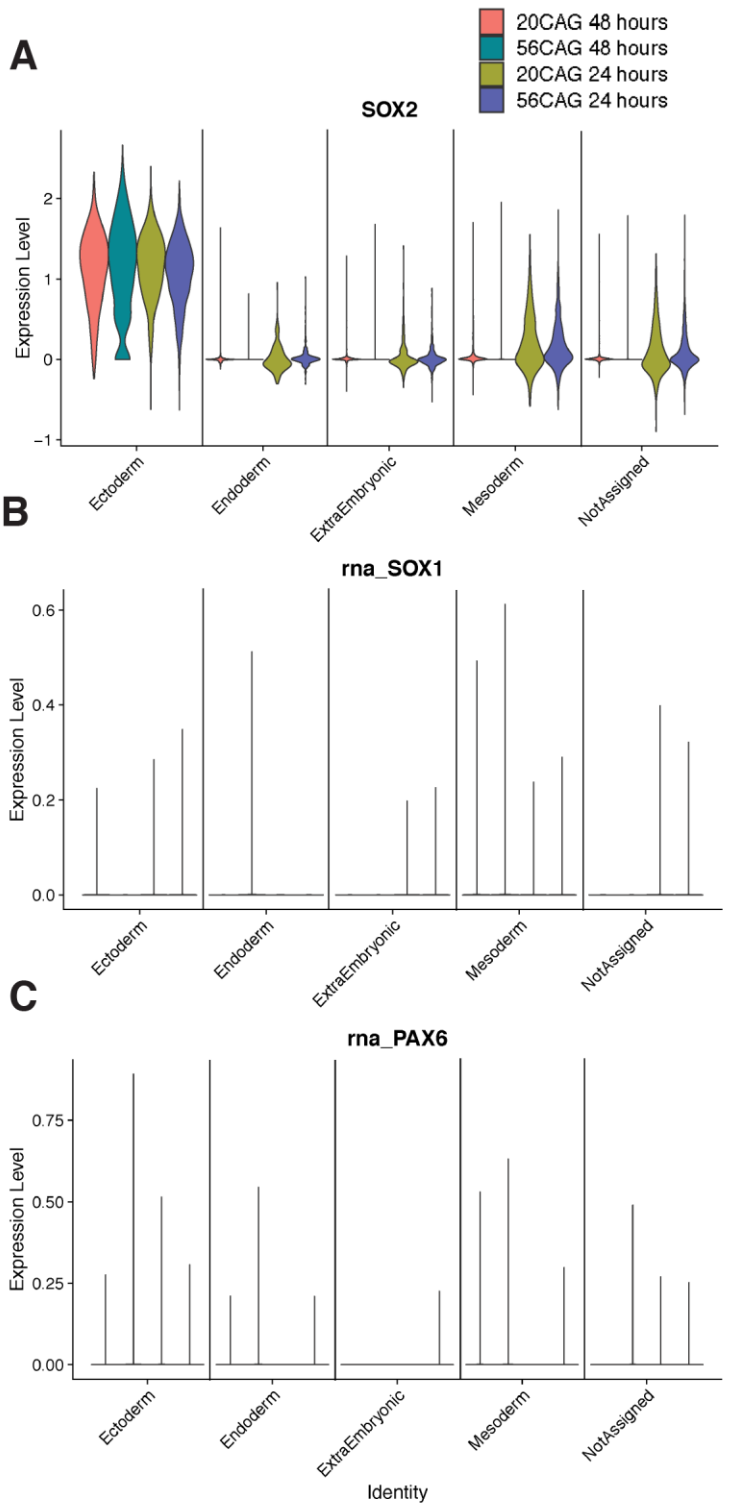

Figure 2: Relative RNA expression levels of SOX2 (A), SOX1 (B) and PAX6 (C) corresponding to different germ layer populations at 24 and 48 hours following BMP4 induction in 20CAG and 56CAG. Data is obtained from single-cell RNA sequencing data set.

"Related to point 4) and 5): whereas it's true that at the culture endpoint there's no obvious difference between HTT+/+ and HTT-/- (Fig 1f,g), the dynamics of reaching this endpoint might be very different. This could be studied by generating HTT-/- cells with the Sox2-mCit construct as done for the CAG-HTT (Fig 1h-j)."

© 2021. Published by The Company of Biologists under the terms of the Creative Commons Attribution License (https://creativecommons.org/licenses/by/4.0/).

We thank the reviewer for the suggestion. Based on data presented in Figure S2b-c, the signaling dynamics of pSMAD1 are not significantly different between HTT+/+ and HTT-/-, whereas 56CAG and 72CAG already demonstrated enhanced pSMAD1 response, which is consistent with the reduction of SOX2 domain in the HD lines. Therefore, rather than analyzing the mCit-SOX2 dynamics, in addressing the reviewer's comment we assessed possible compensatory mechanisms in HTT-/-. Since BMP4 alone is the only condition that does not produce an HTT-/- phenotype, we analyzed NOGGIN reception in 20CAG, 56CAG and HTT-/-. We quantified the distance at which NOGGIN turns pSMAD1 signaling off, and found it to be around 200 µm in both 20CAG and 56CAG. This, however, was strikingly extended in the HTT-/-. This enhanced NOGGIN reception in the HTT-/- can now explain the discrepancies between BMP4 and BMP4+ACTIVIN induced gastruloids in Figures 1f-g and S7a-c. Consistently, blocking endogenous ACTIVIN/NODAL signaling leads to expansion of the SOX2 domain in the HTT-/- compared to HTT+/+. These new results are now presented in Figures 7c-d and S7d-f, and added to manuscript on Pages 15 and 18-19.

"6. The epistasis experiments are important, but very difficult to interpret with the current presentation of the data. All relevant conditions should be presented in the same panel (e.g. those presented in Fig. S3a, c, d; for instance CHIR only). Also, some critical conditions are missing. What is the effect of WNT3a alone? Of Activin alone? Especially the latter is of critical importance, given the effect of CHIR + Activin in HTT-/-. Also, interpretation of these experiments is difficult given the obvious effects of small molecules on germ layer specification in general (independent of the HTT mutations) and may impact cell behavior (proliferation, apoptosis). If certain germ layers are not specified, this will also disrupt the endogenous signals secreted from these germ layers. Also, certainly cells just fill up the space available, even if certain germ layers are not formed? E.g. in BMP4 + IWP2 a similar number of SOX2+ cells may spread over a bigger area in the absence of a mesodermal BRA+ ring. It follows that not only relative ratios of the domains, but also ratios of absolute cell numbers of the different germ layers (and possibly intensities, see point 1 & 4) should be provided."

We thank the reviewer for the constructive criticism, and in agreement with her/his suggestion we have now rearranged the layout of Figure S3 as well as added the missing conditions of WNT3a alone and Activin alone for the HTT-/- in new panels Figure S3a-c. WNT3a alone, similarly to CHIR, produced enhanced endodermal differentiation in both 56CAG and HTT-/- compared to 20CAG. This now substantiates that endogenously produced NODAL leads to increased differentiation in HTT-/- only when BMP4 is bypassed in the signaling hierarchy. Moreover, Activin alone does not induce differentiation in HTT-/-, but maintains pluripotency similar to 20CAG. Additionally, we have now quantified the number of cells in each of the germ layer domains in various conditions as presented in new Figure S3f.

"7. I don't understand the conclusion of the epistasis experiments that "an influence of WNT signaling cannot completely be excluded". In fact, IWP2 leads to a 13% reduction of the center. Moreover, CHIR + Activin leads to a 60% reduction, and the effect of Activin alone has not been addressed (see point 6)."

We respectfully bring to the reviewer's attention that we cannot exclude the contribution of ACTIVIN/NODAL signaling to 13% reduction of the SOX2 domain in the BMP4 + IWP2 condition. Concomitant to this, elimination of ACTIVIN/NODAL signaling leads to diminished SOX2 reduction both downstream of BMP4 and WNT signaling, as demonstrated by BMP4 + SB and WNT + SB or CHIR + SB conditions. Nevertheless, to address ACTIVIN treatment alone for HTT-/-, 20CAG, and 56CAG has now been added to a new Figure S3c. Taken together, our data demonstrate that ACTIVIN signaling is most severely affected by the HTT-CAG expansion in the context of differentiation.

"8. The conclusion that "HTT-CAG expansion affects cell-intrinsic signaling without affecting the transcriptional output… must arise from a non-cell autonomous effect" is based on the analysis of only a few marker genes. Certainly, the possibility that cells transdifferentiate cannot be excluded? Also, I fail to see how this experiment - while elegant! - can reliably make predictions about cell- intrinsic vs non-cell autonomous effects of cells on micropatterns, where cell communication may lead to all kinds of differences in molecular and cellular processes."

We thank the reviewer for describing our experiment as "elegant". Usually, the expression of 3 cell type specific markers is sufficient to diagnose cell types. These results, however, have now also been

© 2021. Published by The Company of Biologists under the terms of the Creative Commons Attribution License (https://creativecommons.org/licenses/by/4.0/).

confirmed by our single cell RNA-seq analysis. Additionally, we agree that differences in cell-cell communication cannot be excluded, but similarly to junctional defects this can only be tested in the context of cell colonies rather than in individual cells, as we defined on Page 10. Inevitably, the consequences of such differences will have an impact on cellular and molecular processes, as the reviewer noted, downstream of cell contacts. We have clarified the sentence on Page 12. Trans-differentiation has been already discussed above on the "Critical Point" of the reviewer.

"9. The authors should clarify the reason for using different concentrations of ACTIVIN & BMP4 in the micropattern (50 resp 100 ng/ml) vs the transwell (10 resp 10 ng/ml) assays? What are the effects of these in WT 2D gastruloids? How could this impact the effects of HTT-CAG and HTT-/-? Are the same effects in the transwell assays observed when using the concentrations of the micropattern assay and vice versa?"

The concentration values used in this study are based on our previous published studies (Warmflash et al., 2015, Etoc et al., 2016, Yoney et al., 2018), and on the timeline of the respective experiment, rather than the culture system as indicated by the reviewer. Lower concentrations (10ng/ml) were used for tracking the short-term response, sufficient to evaluate the activation of signaling effectors, while saturating concentrations (50ng/ml and 100ng/ml respectively) were used for the longer timepoints in the differentiation experiments. In addition, higher ACTIVIN concentration was used for signal activation in CM media condition as described by our previous work (Yoney et al., 2018).

Other suggestions:

"10. The analyses are all based on the analysis of a very limited set of marker genes. The authors could expand with e.g. IFC of early neural markers to test for precocious differentiation and/or transdifferentiation using the Sox2-mCitrine line. Alternatively, with the same line cells could be FACS-purified at different timepoints between 0-40h to analyze transcriptomic changes possibly indicative of precocious and/or transdifferentiation."

We thank the reviewer for the suggestion. We have already addressed the trans-differentiation issue above under "Critical Point" of this reviewer. To enhance this point, we analyzed the expression of early neuronal markers SOX1 and PAX6 as discussed earlier in point 5 and presented in Figure 2 of this rebuttal.

"11. The authors state that "we cannot fully exclude that signal propagation is also shaped by secreted inhibitors". The authors could perform immunolocalization or ISH to test this. Same for the localization of BMP receptors (as mentioned in the next sentence)."

We thank the reviewer for the suggestion. We have now functionally tested the role of NOGGIN as discussed regarding an earlier point of the reviewer and found that while NOGGIN reception is affected by loss of HTT, this is not the case in HTT-CAG expanded cell lines. This is in line with the time-course qPCR experiment in Figure 4e, where we did not detect any difference in the expression of ACTIVIN/NODAL inhibitors LEFTY1, LEFTY2 and CER1 between 20CAG and 56CAG. Indeed, a functional test of the inhibitors would fully exclude their involvement, this falls outside of the scope of the paper at present.
Additionally, we would like to kindly bring to the reviewer's attention that localization of BMP receptors was assessed in Figure 7a.

"12. As described in (a.o.) PMID: 23967334 both CAG-HTT & HTT-KO could upregulate Nodal. Could the authors analyze if this is also the case in their system? If so, how would this affect the patterning of the 2D gastruloids, given the important role of Nodal in this process?"

We thank the reviewer for raising this point. Time-course qPCR analysis and our currently unpublished single cell RNA-seq of NODAL (referred to in points #2 and #5 of Reviewer #1 and point #5 of Reviewer #2) did not reveal any differences at the single cell level between 20CAG and 56CAG as demonstrated by Figure 3 below. This suggests, that the expression of NODAL is not altered by the HD mutation, but rather, it is the enhancement of NODAL reception that leads to patterning defects

© 2021. Published by The Company of Biologists under the terms of the Creative Commons Attribution License (https://creativecommons.org/licenses/by/4.0/).

through the mechanism depicted in Figure 7e.

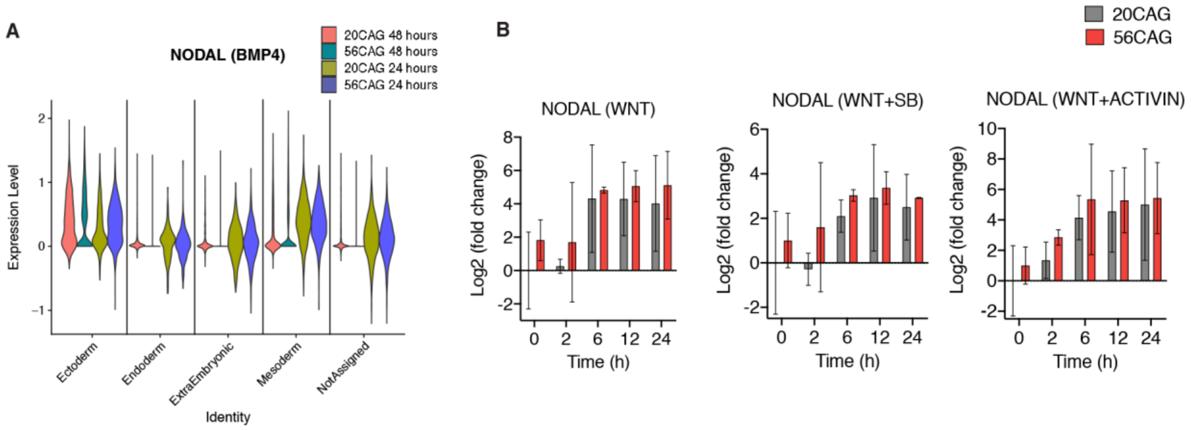

Figure 3. Relative RNA expression levels of NODAL associated with different germ layers following BMP4 induction in 20CAG and 56CAG (A). Time-course qPCR analysis of NODAL following WNT induction with or without ACTIVIN stimulation in 20CAG and 56CAG (B).

"13. Adding a ubiquitous nuclear reporter to the Sox2-mCit line would provide a tool to assess the fate of the Sox2-mCit+ cells."

We thank the reviewer for the suggestion, but it is outside the scope of this study to assess the fate of the SOX2+ cells.

Minor comments:

"1. Although the authors are right that 2D gastruloids are an excellent platform for obtaining quantitative insights, it should be made unequivocally clear that this is a 2D model, not to be confused with 3D gastruloids. The authors should replace "gastruloids" throughout the paper with "2D gastruloids". I also suggest that the authors mention 3D gastruloids (Moris et al., 2020) and acknowledge the limitations of a 2D system to study a 3D process in the discussion of their manuscript."

We thank the reviewer for pointing out the limitation of our study. The discussion has been updated accordingly on Page 16-17 to highlight the limitations of 2D gastruloids, and gastruloids terminology has now been changed to 2D gastruloids throughout the manuscript.

"2. Throughout the figures, color-blind friendly color combinations should be used. In particular the authors should refrain from using red-green combinations (instead used magenta-green or red- blue). In cases where this is not possible (e.g. 3-color combinations), please provide the separate channels in grayscale in a supplemental figure in addition to the merged panel in the main figure."

We thank the reviewer for raising our awareness to this important point. Grayscale images have been added to the supplementary material as new Supplementary Figure 8 for panels of relevance.

"3. Although the manuscript is in general clearly and well-written, there are multiple grammar and spelling errors throughout the manuscript. Please carefully reassess the text to correct these."

We thank the reviewer for bringing this to our attention. We have now corrected typos and grammatical mistakes.

© 2021. Published by The Company of Biologists under the terms of the Creative Commons Attribution License (https://creativecommons.org/licenses/by/4.0/).

"4. The sample sizes are not consequently provided in all of the figure legends (e.g. Fig 1B). Please ensure that this is the case where relevant. In particular, sample sizes are not provided in the imaging panels. How many times was the same (or similar) result achieved? Please add an n/N to the figures ("the shown phenotypes were observed in n colonies out of N colonies in X independent experiments")."

We should have been more clear, and would like to respectfully bring to the reviewer's attention that representative imaging data is displayed for all quantification and sample sizes (e.g. number of colonies / number of images analyzed) were described for each corresponding graph. Subsequently, the number of independent experiments is reported at the end of each figure legend.

"5. The resolution of some of the imaging data is very low (e.g. Fig 1C). Please provide higher resolution data."

Higher resolution images were added to Figure 1.

"6. Could the authors explain why in Fig 1E there's a clear effect in the 43CAG and 48CAG condition whereas this is supposed to represent late onset Huntington's Disease?"

It is the strength of our highly quantitative assay that it is sensitive enough to detect even small changes elicited by the HTT-CAG expansion, however it is a reductionist approach that cannot account for features of the embryo such as extraembryonic tissues and the uterine lining. It is not fully understood how this and other compensatory mechanisms shape the development of HD gene carriers, but it is clear that abnormalities caused by even lower CAG-lengths (39-42CAG) could be detected in the fetal cortex as early as gestational week 13 (Barnat et al., 2020).

"7. Fig. 2f: Are these values normalized for DAPI intensity? It's (very) hard to appreciate the quantified differences based on the stainings in Fig. 1d."

The results are normalized for DAPI (we believe that the reviewer is actually referring to Fig 2d). We would kindly like to bring to the reviewer's attention that in Fig. 2e we better resolve the differences plotted in Fig. 2f, where we also perform the statistical analysis.

"8. Fig. 4a: where are the raw data of the other conditions (of which a quantification is provided in Fig 4b)?"

All data sets will be made publicly available upon acceptance of the paper.

9. In Fig 7a & 7c there are mysterious colored rectangles on top of the figure where most likely the coloring legend of the staining should be presented. Same for Fig 6a on the left side.

The rectangles must have replaced the color legend during the compression of the original figure files. We apologize for the error.

10. How physiologically relevant is the observed expansion of the endodermal population? Are there any abnormalities in endoderm-derived associated with Huntington's disease?

To our knowledge, no *in vivo* studies have investigated the status of endodermal populations in human fetal samples, but ectopic ACTIVIN/NODAL signaling has been detected in the mouse consistent with the endodermal expansion (Woda et al., 2005). Interestingly, inhibition of MYOSTATIN/ACTIVIN signaling in HD mice reduced peripheral symptoms such as body weight loss, muscular atrophy and weakness (Bondulich et al., 2017). It would be of interest to evaluate what organ systems are affected by enhanced ACTIVIN signaling in HD patients.

11. "Impaired spatial restriction to ACTIVIN signaling observed in HTT-CAG expanded colonies suggest a failure of this mechanism…". Do cells first transdifferentiate (if they do?) before or after spatial restriction of ACTIVIN is impaired? In other words, cause or consequence?

We would like to kindly bring to the reviewer's attention that the pluripotency status of our cell lines was thoroughly characterized in our previous publication (Ruzo et. al., 2018) and we find the 1 hour

© 2021. Published by The Company of Biologists under the terms of the Creative Commons Attribution License (https://creativecommons.org/licenses/by/4.0/).

Activin stimulation too short of a timeframe to allow for trans-differentiation, as stated above in the Critical point of the reviewer.

12. I don't understand why "in HTT-/- line ZO-1 expression was selectively absent in cells responding to apical ACTIVIN stimulation". Would tight junction integrity not lead to ACTIVIN freely diffusing to the apical side by impairing barrier function, thus also affecting other cells?

We agree with the reviewer that apically presented ACTIVIN might "leak" to the basal compartment with impaired tight junction integrity. The measured timeframe however, 1 hour in this case, would not be sufficient for the diffusion and subsequent signal activation at the cell density tested.

13. "In human gastruloids, this led to enhanced mesendodermal differentiation and as a consequence a smaller ectodermal domain". In the absence of live tracking of cells labelled with mesendodermal and ectodermal markers there's no unequivocal evidence that this is the cause-consequence sequence.

We have corrected the sentence as per the reviewer's suggestion to remove a direct connection between cause and consequence.

14. "Since the 56CAG extended line phenocopies…this observation confirms that cellular polarization is compromised in the HTT-CAG expanded background, even though expression of ZO-1 remains intact." There could be other explanations, e.g. different developmental trajectories for HTT-CAG and HTT-/- to reach the same endpoint, with compromised polarization being important for HTT-/- phenotype, but ACTIVIN triggering transdifferentiation potential in the HTT-CAG line.

We would like to kindly bring to the reviewer's attention that we detected ectopic ACTIVIN signaling at 1 hour following stimulation. At this stage the cells are still considered pluripotent (Yoney et al., 2018) and there is no apparent difference between the genotypes in their pluripotency (Ruzo et al., 2018). If trans- differentiation occurs, that must happen downstream of pluripotency and impaired cellular polarization.

15. The authors should remove the last half-sentence "could shift the clinical focus….during the prodromal phase". This is unnecessary and speculative at best. If the authors want to keep the sentence they should provide a clear example of how such prodromal clinical intervention could look like.

We fully agree with the reviewer that our study cannot be directly translated clinically, therefore it would be farfetched to delineate a concrete clinical strategy. With that in mind, we deleted the sentence and softened our statement on Page 19.

16. Fig S1c: why is the effect of 53CAG more severe than 73CAG?

We agree with the reviewer that we should have been more clear. The experiment in Figure S1a-d was performed on HD iPSCs of non-isogenic origin, therefore genetic modifiers are likely to play a role in establishing the phenotype. We consider it a strength of our system to sensitively detect the presence of such modifiers.

17. Fig S1f: HD-C1 and HD-C2 vs Non-HD also seem significant? Please provide stats for those comparisons. If difference is significant, what could be the reason for that?

We agree with the reviewer that there is statistical significance between HD-C1 and HDC-2 vs Non-HD ($p=0.03$ and $p=0.02$ respectively), however this is much smaller than the difference detected between Non- HD vs HD or HD-C vs HD (p values and method described in the figure legends) and it could be attributed to the non-isogenic heterogeneity innate to the iPSCs. Nonetheless, it is outside the scope of this study to analyze this aspect of the experiment more deeply.

© 2021. Published by The Company of Biologists under the terms of the Creative Commons Attribution License (https://creativecommons.org/licenses/by/4.0/).

We want to thank Referee #2, as well as Referee #1, again, for taking the time to thoroughly evaluate our manuscript. Their suggestions and criticisms have significantly improved the quality of our study and our manuscript, which we hope they will now find acceptable for publication in *Development*.

Second decision letter

MS ID#: DEVELOP/2021/199513

MS TITLE: Huntingtin CAG expansion impairs germ layer patterning in synthetic human 2D gastruloids through polarity defects.

AUTHORS: Szilvia Galgoczi, Albert Ruzo, Christian Markopoulos, Anna Yoney, Tien Phan-Everson, Shu Li, Tomomi Haremaki, Jakob J. Metzger, Fred Etoc, and Ali H Brivanlou
ARTICLE TYPE: Research Article

I am happy to tell you that your manuscript has been accepted for publication in Development, pending our standard ethics checks.

Reviewer 1

Advance summary and potential significance to field

NA

Comments for the author

The revision by Galgoczi et al has addressed most of the issues and is now appropriate for publication.

Reviewer 2

Advance summary and potential significance to field

In this manuscript, the authors use 2D "gastruloids" - geometrically confined micropatterned cultures of hPSCs - to demonstrate that HTT mutations, both CAG expansion repeats (CAG-HTT; causal to Huntingon's Disease) and HTT-KO, cause severe disruption of the self-organized concentric circles representing the germ layers. As I've stated in my first review, the authors make elegant use of the 2D gastruloid model to provide further insights into the mechanisms governing HD in HTT-CAG and HTT-/- conditions. The manuscript has been significantly revised including some important new experiments.

Although not all of my points have been addressed, I appreciate the efforts of the authors that vastly improved the paper. I therefore recommend publication in Development, pending the following revisions:

Comments for the author

1. As requested, the authors have now analyzed apoptosis and quantified cell density across conditions (Fig S7G-H). These data leads them to conclude that
"we did not detect decreased cell density or enhanced apoptosis in 56CAG or HTT-/- compared to 20CAG". However, this is not backed by statistical analysis of the data and in fact eyeballing of the individual datapoints (Fig S7H) suggests that the differences (at least 20CAG vs HTT-/- for cell numbers & 56CAG vs HTT-/- for apoptosis) are statistically significant. This should be tested & if this is the case statements in the text re this experiment should be adjusted.

© 2021. Published by The Company of Biologists under the terms of the Creative Commons Attribution License (https://creativecommons.org/licenses/by/4.0/).

2. The argumentation the authors provide re the possibility of precocious and trans-differentiation is useful and important, and should be included in the text. That said, I do not fully agree with the reasoning. Upon loss of SOX2 (between 20h & 30h for 72CAG) there would still be a window of 18-28h to transdifferentiate, and this is not in any way addressed in the revision. If this is outside the current scope, this limitation should be clearly stated.

3. In addition to the previous comment: the reviewer figures with the scRNA-seq data are important to back some of the statements, but this makes it problematic that these will not be included in the final version of the manuscript. It would be an easy experiment to add immunostainings of PAX6 & SOX1 on a similar timecourse of the SOX2-Citrine reporter line as presented in Figure 1I. This analysis should be complemented with 72CAG, since Fig 1I suggests the clearest effect for this condition - currently not included in scRNA-seq data.

4. The authors point me to Fig S2A for cell density quantifications, but this is for pSMAD, and NOT for the different germ layers as requested. Please provide.
The authors should also provide statistical analysis for Fig S2E, given that total cell numbers appear significantly lower in 72CAG compared to 20CAG (as I pointed out in my previous review, Fig 1B seems to suggest the same for culture endpoint).

5. In Fig S3F a quantification of ALL conditions should be provided, not just selected.

6. My main point 4 has not been addressed. I asked time-resolved quantification of the signal intensity and the absolute number of SOX2-Citrine+ cells, but the authors point me to pSMAD and overall quantifications.

7. From Reviewer Fig 3A the authors draw the conclusion that Nodal expression is not increased in 56CAG but the data suggest otherwise? By eyeballing I'd say expression is lower at 48h and higher at 24h. Please quantify. In fact, it would be very informative to add qPCR data to the paper, given that the scRNA-seq data will not be included in the manuscript.

8. Quantifications S1G: please also provide absolute numbers (like in S3F) for all conditions.

9. The authors state that the raw imaging data for Fig 4B will be released upon publication, but it seems illogical that only for the control condition these are provided in the paper (Fig 4A). The authors should add a similar panel for 56CAG + BMP4

10. Please indicate clearly where isogenic and where non-isogenic cell lines are used since as you state genetic background may interact with mutation effects.

© 2021. Published by The Company of Biologists under the terms of the Creative Commons Attribution License (https://creativecommons.org/licenses/by/4.0/).
